# Growth rate is a dominant factor predicting the rhizosphere effect

**DOI:** 10.1038/s41396-023-01453-6

**Published:** 2023-06-15

**Authors:** José L. López, Arista Fourie, Sanne W. M. Poppeliers, Nikolaos Pappas, Juan J. Sánchez-Gil, Ronnie de Jonge, Bas E. Dutilh

**Affiliations:** 1 Theoretical Biology and Bioinformatics, Department of Biology, Science for Life, Utrecht University, Utrecht, The Netherlands; 2 Instituto Andino Patagónico de Tecnologías Biológicas y Geoambientales, Bariloche, Rio Negro Argentina; 3 Institute of Biodiversity, Faculty of Biological Sciences, Cluster of Excellence Balance of the Microverse, Friedrich Schiller University Jena, Jena, Germany; 4 Plant-Microbe Interactions, Department of Biology, Science for Life, Utrecht University, Utrecht, The Netherlands

**Keywords:** Metagenomics, Microbiome, Microbial ecology, Bacterial genetics, Metagenomics

## Abstract

The root microbiome is shaped by plant root activity, which selects specific microbial taxa from the surrounding soil. This influence on the microorganisms and soil chemistry in the immediate vicinity of the roots has been referred to as the rhizosphere effect. Understanding the traits that make bacteria successful in the rhizosphere is critical for developing sustainable agriculture solutions. In this study, we compared the growth rate potential, a complex trait that can be predicted from bacterial genome sequences, to functional traits encoded by proteins. We analyzed 84 paired rhizosphere- and soil-derived 16S rRNA gene amplicon datasets from 18 different plants and soil types, performed differential abundance analysis, and estimated growth rates for each bacterial genus. We found that bacteria with higher growth rate potential consistently dominated the rhizosphere, and this trend was confirmed in different bacterial phyla using genome sequences of 3270 bacterial isolates and 6707 metagenome-assembled genomes (MAGs) from 1121 plant- and soil-associated metagenomes. We then identified which functional traits were enriched in MAGs according to their niche or growth rate status. We found that predicted growth rate potential was the main feature for differentiating rhizosphere and soil bacteria in machine learning models, and we then analyzed the features that were important for achieving faster growth rates, which makes bacteria more competitive in the rhizosphere. As growth rate potential can be predicted from genomic data, this work has implications for understanding bacterial community assembly in the rhizosphere, where many uncultivated bacteria reside.

## Introduction

Soils represent the most complex and diverse microbiomes in the world. A notable component of these is the rhizosphere, comprising the soil region near plant roots, which are influenced by root exudates and rhizodeposition [1]. Rhizosphere microbiomes assemble by enriching a subset of the microbiota present in the surrounding bulk soil, which may pose beneficial, neutral, or detrimental effects on the host plants [1, 2]. The changes in microbial community toward the rhizosphere, known as the “rhizosphere effect” [3], involves a decrease in species richness imposed by stronger selection [4]. Rhizosphere communities display significant variation across experimental settings, exhibiting distinctive or overlapping microbial composition compared to the bulk soil [4–10]. While methodological differences between these studies cannot be ruled out, including biases during rhizosphere microbiome isolation, there may also be physiological factors that influence the rhizosphere effect [3]. On the plant side, the rhizosphere effect may be influenced by the host plant species [11], the stage of the plant life cycle [12], or the location on the root [8, 13].

Understanding the biological factors driving rhizosphere community assembly has been a complex and intricate endeavor [3]. Approaches to studying rhizosphere-associated traits have included analyzing changes in bacterial composition or function between roots and soils [14–19]. A comparative analysis of thousands of genomes from isolates obtained in plant-associated and non-plant-associated environments has identified genomic traits associated with plant colonization [14], but analysis of unculturable bacteria was previously lacking.

Besides taxonomic or functional changes in the rhizosphere microbiome, growth rate potential has been suggested as an ecological indicator of microbial lifestyle in the soil [20]. Copiotrophs, adapted to high nutrient conditions with faster growth rates, are enriched in soils with abundant labile organic substrates (i.e., glycine, sucrose), while oligotrophs, adapted to low nutrient conditions with slower but more efficient growth, are enriched in soils containing recalcitrant chemicals (i.e., cellulose, lignin, or tannin–protein) [21]. Agricultural inputs of nitrogen and phosphorus have also been associated with increased relative abundance of copiotrophic bacteria [22]. These observations suggest that plant root exudates may contribute to the selection of copiotrophs in the rhizosphere, playing a role in establishing the rhizosphere effect.

Recently, a model based on genomic data has enabled estimating bacterial growth rate potential without the need for culturing [23]. This model takes into account codon usage bias, codon usage pattern consistency in highly-expressed genes, and genome-wide codon pair bias, to estimate a minimum doubling time that can be used to classify microbes as fast- or slow-growing [23]. Growth rate potential has been explored in various biomes, including the nutrient-rich human gut and in oligotrophic marine systems, where it was found that community-wide average growth rate potential decreased with depth, likely due to a decrease in nutrient availability [24, 25]. To study how growth rate potential may contribute to the rhizosphere effect, here we analyzed 460 rhizosphere and 232 bulk soil 16S rRNA gene datasets comprising 84 paired rhizosphere and bulk-soil datasets from 18 different plant genotypes, a set of previously analyzed isolate genomes from plants and soils [14], and MAGs from 501 rhizosphere and 620 soil full metagenomes from diverse studies.

## Methods

### Matching rhizosphere and bulk soil datasets in 16S rRNA gene datasets

We identified studies in the MGnify database [26] where both rhizosphere and bulk soil samples were available with sufficient sequencing depth (>10,000 reads/sample, Supplementary Table 1). We downloaded BIOM files of SSU rRNA amplicon sequence variant (ASV) counts and taxonomic assignments and prepared genus-rank abundance matrices. Using DESeq2 [27], we identified genera enriched in the rhizosphere or bulk soil (adjusted *p* < 0.05, Benjamini-Hochberg FDR, log-2 fold change (L2FC) > 1 for soil-enriched genera, L2FC < −1 for rhizosphere-enriched genera). We analysed genera because ASVs often cannot be classified at the species level, and there is a strong conservation in growth rate potential at the genus rank [23, 28].

### Minimal doubling time predictions

We estimated bacterial growth rate potential using the gRodon R package and EGGO database, which includes predicted minimum doubling times (PMDTs) for over 217,000 prokaryotic genomes, MAGs, and single-amplified genomes (SAGs) [23]. To avoid unreliable predictions, particularly in slow-growing organisms where codon usage bias patterns may be lost [23], we computed the median PMDT (mPMDT) for each identified genus from the affiliated genomes in the EGGO database. We estimated PMDTs for MAGs and isolate genomes using gRodon 1.0.0 [23]. Ribosomal protein-coding genes were identified by searching “rps”,”rpm”,”rpl” terms in the eggNOG annotation file, and used as highly expressed reference genes for gRodon predictions [29], which was run using “partial” mode, and “vs” training set. Following Weissman et al. [23] we classified copiotrophs or oligotrophs as having PMDTs of <5hs and ≥5hs, respectively, but we also analyzed other cutoffs. Copiotrophs tend to have a higher maximal growth rate and are more responsive to carbon sources, but use resources less efficiently than oligotrophs, which in turn grow relatively slowly and prefer nutrient-poor environments with low energy flows [28]. We use the terms copiotrophs/oligotrophs and fast-/slow-growing bacteria interchangeably, although we acknowledge that the former are also associated with differences in resource utilization, which we did not analyze.

### Functional and phylogenetic annotation of MAGs

We obtained high- and medium-quality (HQ/MQ) rhizosphere and soil MAGs (≥50% completeness, <10% contamination [30]) from IMG/M [31]. IMG/M pipeline includes Metabat v2:2.15 [32] and checkM v1.1.3 [33]. We extracted contigs and functional annotations for each MAG and created presence/absence matrices of Clusters of Orthologous Groups (COGs), KEGG Orthology (KO), and Protein Families (Pfam). For gRodon predictions on MAGs and isolate genomes, nucleotide and protein sequences for genes were predicted using Prodigal version 2.6.3 [34] and functions were annotated using eggNOG mapper 2.1.3 [35]. We further grouped functions into broader modules with DRAM 1.4.6 [36] and expanded DRAM to a total of 445 categories including KEGG modules, hydrolytic enzymes, and flagellar assembly (Supplementary Table 2). Module completeness was calculated as in DRAM. To place MAGs in the context of known strains, we generated a maximum-likelihood phylogeny from concatenated GTDB marker genes, using GTDB-Tk 1.3.0 with database 95 ([37], gtdbtk identify, align, and infer).

### Mapping whole metagenomic samples from paired rhizospheres and their bulk soils to our MAGs collection

We downloaded paired rhizosphere and bulk soil samples from *Arabidopsis thaliana*, *Cucumis sativus*, and *Triticum aestivum* (Supplementary Table 3) and performed a QC quality filtering step as implemented in ATLAS [38]. QC-filtered reads were then mapped to our collection of MAGs (see above) using BBMap [39] with default parameters. To achieve this, we created a reference database of all the MAG contigs concatenated into a single sequence, including 200 N characters between each contig. Bam files were sorted, and horizontal coverage was obtained for each MAG using samtools [[40], sort and coverage commands]. The number of mapped reads to each MAG were used as input for differential enrichment using DESeq2 [27], and significantly enriched MAGs in rhizospheres or bulk soil samples were identified using adjusted *p* < 0.05.

### Phylogeny-aware functional enrichment analyses

To identify functions associated with rhizosphere or soil bacteria, MAGs were labeled according to the sample from which they were recovered (rhizosphere or soil, Supplementary Table 4). Phylogenetic Generalized Linear Models (PhyloGLMs) [41] were used for functional enrichment analysis using presence/absence of functional categories (KO, COG, or Pfam) as independent variables to predict rhizosphere/soil association. Similar models were generated to predict functions associated with copiotrophs (PMDT<5hs) or oligotrophs (PMDT≥5hs, Supplementary Table 5). We corrected *p* values with Benjamini-Hochberg FDR and used adjusted *p* < 0.05 to consider significantly enriched functions. The broader DRAM categories were also used to predict rhizosphere/soil and copiotroph/oligotroph associations, using a completeness cutoff of 50% to determine the presence/absence of modules. These analyses were performed using the phylolm R package v. 2.6.2 [41].

### Machine learning models

Binary matrices contained 6707 MAGs (3692 rhizosphere and 3015 soil) and 8680, 4841, 9132, and 445 binary features for KO, COG, Pfam, and DRAM respectively. To classify MAGs as rhizosphere- or soil-associated, we used Random Forest (RF) and Gradient Boosting Classifier (GBC) models trained on a binary target label vector based on MAG origin, utilizing the scikit-learn Python package (https://scikit-learn.org/). We employed 5-fold cross-validation and tested RF hyperparameters including number of trees (n_estimators), maximum number of features per node (max_features), maximum depth of trees (max_depth), maximum leaf nodes in the trees (max_leaf_nodes), minimum number of samples to create a leaf node (min_samples_leaf), and minimum number of samples to generate a split (min_samples_split). For GBC models, we evaluated different settings for n_estimators, max_depth, and the learning rate (learning_rate). We also obtained Gini feature importances, which measure the relative accumulation of impurity decrease for each feature in the model. Functional connections between the most important and significant COGs were analyzed with STRING 11.5 [42].

## Results and discussion

### Rhizosphere bacteria have shorter predicted doubling times than soil bacteria

**Fig. 1:**
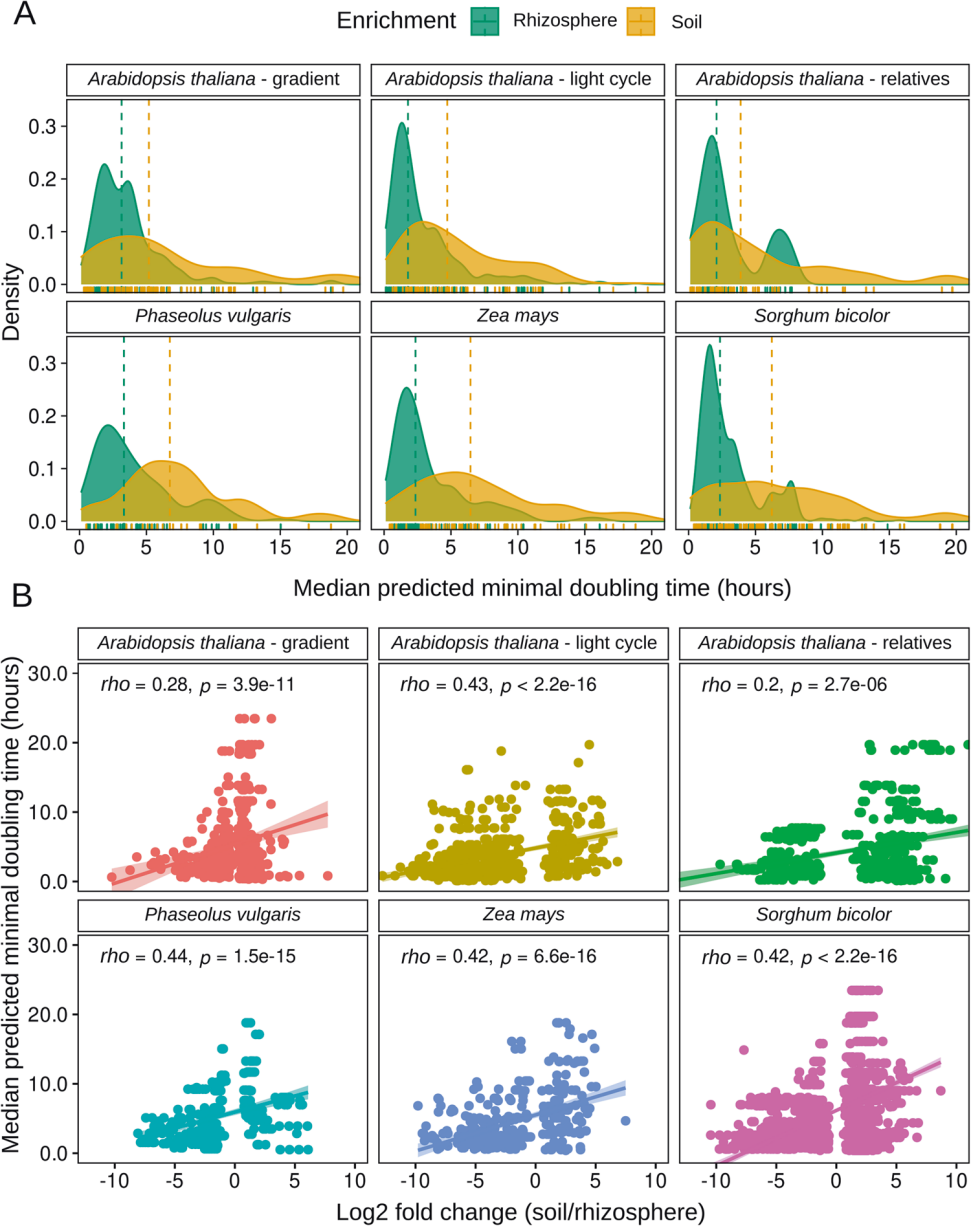
Median predicted minimum doubling times (mPMDT) of bacteria enriched in rhizospheres are lower than those in associated bulk soils. DESeq2 log2 fold-change was used to categorize bacteria as being enriched in the rhizosphere (L2FC < −1) or soil (L2FC > 1). Soil-enriched bacteria tend to have a higher mPMDT. **A** Density distribution of bacteria enriched in the rhizosphere or soil. **B** A positive correlation exists between soil enrichment and mPMDT, i.e. the rhizosphere contains faster growers than the bulk soil.

To investigate whether growth rate potential predictions correlate with rhizosphere enrichment, we re-analyzed previously published metacommunities. These included rhizospheres and associated bulk soil microbiomes from 18 different plant genotypes and conditions such as: *Arabidopsis thaliana* ecotypes and sister species [9], *A. thaliana* Col-0 ecotype under light-dark cycles [43], wild and modern accessions of *Phaseolus vulgaris* (common bean) [44], *Zea mays* grown in different crop rotations [45], *Sorghum bicolor* under drought stress and control conditions, at different timepoints in their lifecycle [46], and *A. thaliana* Col-0 sampled along a bulk soil-to-rhizosphere gradient [47]. First, we identified genera enriched in rhizosphere or bulk soil, and compared the median predicted minimal doubling time (mPMDT) associated with these genera (See Methods). Growth rate potential is generally conserved below the genus level [23, 48]. Thus, by grouping ASVs to genus level we were able to expand the number of ASVs analysed to include all those with missing species-level taxonomy affiliations. This allowed us to compare the mPMDT of rhizosphere-enriched to soil-enriched genera (see Materials and Methods), which revealed that rhizosphere-enriched genera have on average faster growth rates (lower mPMDT) than soil-enriched genera (Fig. 1A-B).

Analyzing diverse datasets in more detail allowed us to assess whether this selection was consistent across different experimental conditions. First we observed this general trend in different species of the same plant genus (*Arabidopsis*), in modern and wild accessions of a same species (*P. vulgaris*), and in different plant hosts (*A. thaliana*, *P. vulgaris*, *S. bicolor*, and *Z. mays*) (Supplementary Figs. 1–3), showing that although plant hosts induce specific compositional shifts in the rhizosphere microbiomes [11], faster growth rates to colonize rhizosphere seems to be a common factor. Second, although the host’s circadian rhythm induces changes in the rhizosphere microbiome and the soil organic matter composition [43], it does not affect the rhizosphere-enrichment of fast-growing bacteria (Supplementary Fig. 2). Third, different soil conditions, such as crop rotations and drought stress do not modify this general trend either, as shown here in *Z. mays* and *S. bicolor* ([45, 46], Supplementary Fig. 1,3). Fourth, in a study where a gradient from bulk soil to the rhizoplane was experimentally dissected and analyzed separately [47], we observed that copiotrophs increased gradually as samples were taken closer to the root (Supplementary Fig. 4). Thus, we observed that the trend for fast-growing bacteria to colonize the rhizosphere was consistent and independent of the plant species or ecotype, soil type, or experimental condition.

When we observed exceptions to this pattern of fast growers enriched in the rhizosphere, i.e., copiotroph bacteria that were enriched in soils, these were mostly among genera affiliated with *Firmicutes* (Supplementary Figs. 1–3). Most bacteria affiliated with *Firmicutes* are among the fastest growers in the bacterial tree [23, 49], thus it may be harder to prove this trend in the narrower mPMDT distributions present in this particular phylum.

When analyzing the correlations between the L2FC enrichment scores and the mPMDT by the four most abundant phyla in our 16S rRNA data (*Actinobacteria*, *Bacteroidetes*, *Firmicutes*, and *Proteobacteria*), we found a significant correlation between mPMDT and rhizosphere enrichment in taxa affiliated with *Proteobacteria* in all projects (Supplementary Table 1). When merging all samples from different projects, enrichment of the copiotrophs was significant for genera affiliated with *Proteobacteria, Actinobacteria*, *Bacteroidetes*, *Acidobacteria*, and *Verrucomicrobia*, but not with *Firmicutes* (Supplementary Fig. 5). Thus, 16S rRNA gene data shows that fast-growing genera are preferentially enriched in rhizospheres compared to bulk soils in members of these main bacterial phyla.

### Bacterial genomes confirm copiotrophs are predominant in the rhizosphere

To further analyze changes in growth rate potential distributions using genome sequences of isolated bacteria, we estimated PMDTs in genomes from cultured bacteria isolated from plant, non-plant, root/rhizosphere, and soil biomes reported in Levy et al. [14], including 3270 genomes classified into taxonomic groups *Actinobacteria* groups 1 and 2 as defined by the authors, *Alphaproteobacteria*, *Bacillales*, *Bacteroidetes*, *Burkholderiales*, *Pseudomonas*, and *Xanthomonadaceae* [14]. In *Alphaproteobacteria* and *Bacteroidetes*, bacteria affiliated with these phyla isolated from rhizoplane and endophytic compartments (root associated, RA) have lower PMDT than those isolated from soils (Supplementary Fig. 6). A similar observation extended to *Actinobacteria*_2, *Alphaproteobacteria*, *Bacillales*, and *Bacteroidetes*, when comparing bacterial genomes isolated from plant niches, including rhizospheres (plant associated, PA) and from non-plant environments (NPA, which includes both soils and other environments like marine or clinical). Thus, although isolation protocols tend to select for copiotrophs [23], we still observed lower PMDTs in cultured isolates from plant environments compared to those obtained from soil.

**Fig. 2:**
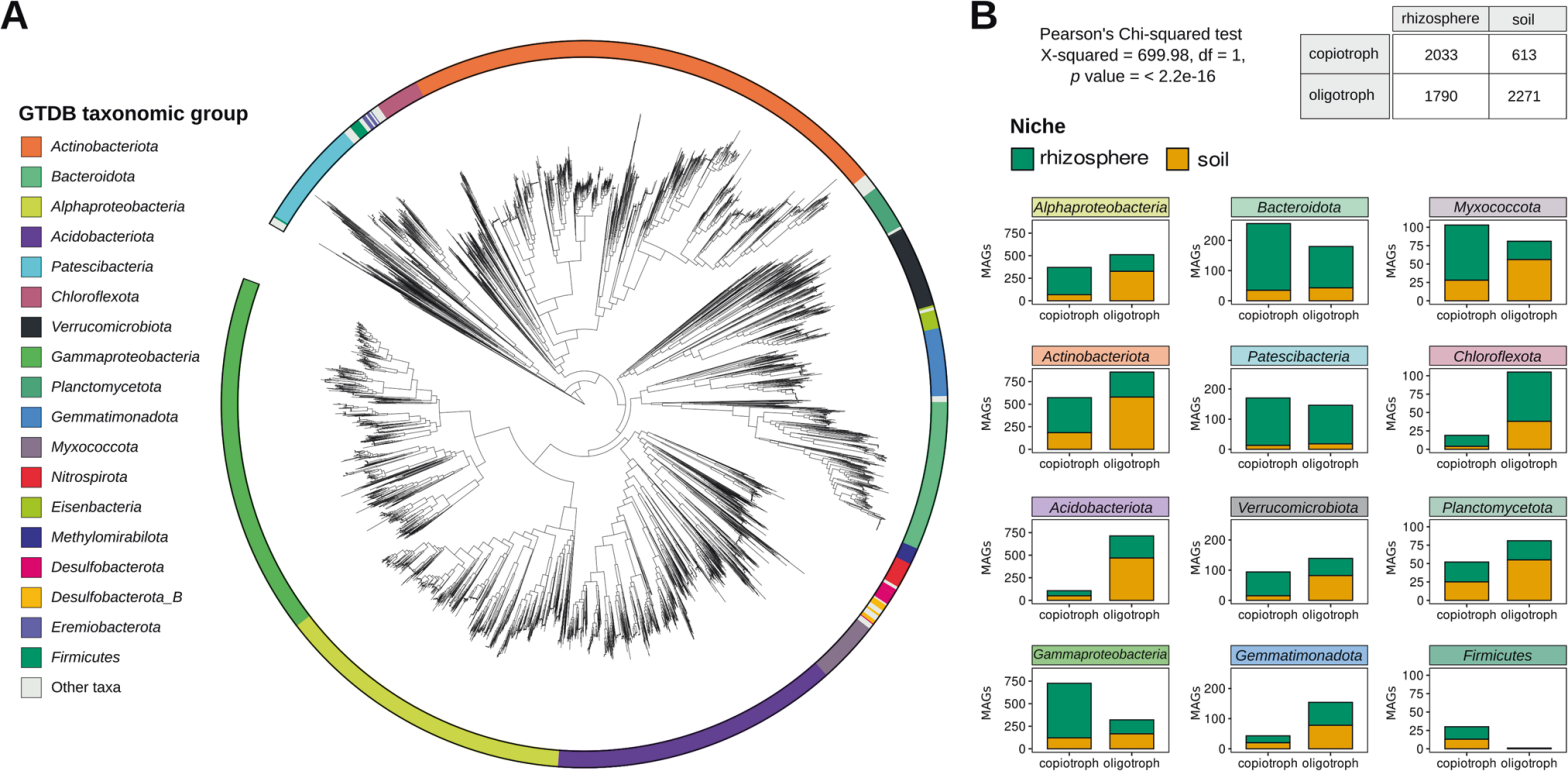
MAGs taxonomy, niche, and growth rate status. **A** Unrooted maximum-likelihood phylogenetic tree inferred from multiple sequence alignments of GTDB bacterial marker genes from MAGs. The tree was generated with GTDB-Tk and displayed using iTol [80]. **B** MAGs are classified according to their isolation biome and growth rate status (copiotroph or oligotroph) for each of the main GTDB taxa. MAGs included members affiliated with the *Actinobacteriota* (1453), *Proteobacteria* (including 1063 MAGs from *Gammaproteobacteria* and 869 MAGs from *Alphaproteobacteria*), *Acidobacteriota* (850), *Bacteroidetes* (440), *Patescibacteria* (322), *Verrucomicrobiota* (248), *Gemmatimonadota* (200), *Myxococcota* (187), *Planctomycetota* (133), *Chloroflexota* (127), *Nitrospirota* (76), *Eisenbacteria* (56), *Methylomirabilota* (51), *Desulfobacterota* (47), *Desulfobacterota*_B (45), and *Firmicutes* (36), among others (Supplementary Table 4).

To avoid any possible biases associated with bacterial cultivation, we extended our genomic analyses to MAGs. We downloaded 501 whole-metagenome rhizospheric samples from different plants and 620 whole-metagenome soil samples from different biomes. We then recovered 3679 high-quality and medium-quality (HQ/MQ) bacterial MAGs from rhizospheres and 2784 HQ/MQ bacterial MAGs from soils (see Fig. 2, Materials and Methods, Supplementary Table 4). PMDT could be predicted for 6355 of these MAGs, of which 2629 were copiotrophs, 3726 were oligotrophs, while 3652 were obtained from rhizosphere samples and 2703 were obtained from soils. A chi-squared test shows that PMDT and the isolation niche are significantly associated (Pearson’s Chi-squared, *p* < 2.2e-16). For a MAG, being copiotroph is significantly associated with colonizing the rhizosphere (PhyloGLM, estimate: 1.47, *p* value: 2e-16).

**Fig. 3:**
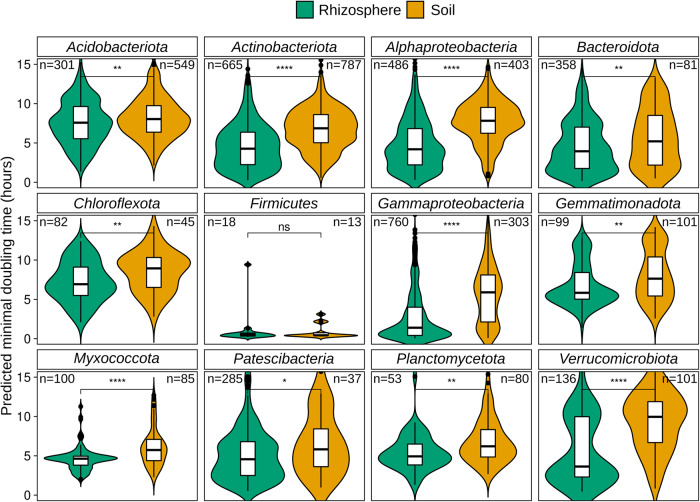
PMDT in MAGs from rhizosphere and soil metagenomes. Distributions of predicted minimal doubling times in MAGs from rhizosphere and soils were compared with Mann–Whitney test (ns: *p* > 0.05, *p < =0.05, **p < =0.01, ***p < =0.001, ****p < =0.0001).

The analysis of MAGs allowed us to compare predicted growth rates of unculturable bacteria across a wide range of taxonomic groups (Supplementary Table 5). As shown in Weissman et al. [23], collections of isolates fail to capture the most slowly growing members of the communities, when compared to MAGs or SAGs from the same environments. Despite being obtained from diverse metagenomes and belonging to different plants and soils, our predictions of PMDT revealed that MAGs obtained from rhizosphere metagenomes have significantly lower PMDT than MAGs obtained from soils across all major taxonomic groups except for MAGs affiliated with *Firmicutes*, which have an extremely low and narrow PMDT distribution (Fig. 3). These results also support the use of finer taxonomic levels (e.g., genus instead of phylum) to classify microbes based on their life history strategies, as reported by other authors [28, 49]. Furthermore, the rhizosphere niche appears to preferentially select fast-growing microbes capable of taking advantage of the higher nutrient concentrations from root exudates. We suggest that the presence of fast-growing bacteria in the rhizosphere may indicate a copiotrophic lifestyle, with the enrichment likely explained by the higher nutrient concentrations in the rhizosphere compared to soil. We acknowledge the need for more detailed analyses of resource use, growth yield, and growth rates for a more comprehensive ecological understanding [28, 49–52].

We then set out to investigate the relative enrichment of fast-growing MAGs in the rhizosphere. Although obtaining a reasonable number of HQ/MQ MAGs from paired rhizosphere and their corresponding bulk soils samples is challenging due to the overwhelming diversity of these biomes, we devised a strategy to quantify the relative enrichment using whole metagenome data, which further extends the results observed with amplicon data. We mapped reads belonging to paired rhizosphere-bulk soil samples from three plant species (*Arabidopsis thaliana* [53], *Cucumis sativus* [54], and *Triticum aestivum* [55]) to the collection of MAGs presented above. This approach assumes that the paired samples contain sequences from related MAGs to those in our collection and succeeds in revealing the same pattern observed throughout this work. At a horizontal coverage cutoff of >20%, we identified between 100–400 MAGs per sample, allowing us to carry out a differential enrichment analysis. The analysis revealed that PMDTs in rhizosphere-enriched MAGs were significantly lower than those from MAGs associated with the paired bulk soil samples (Supplementary Fig. 7).

### MAGs provide a catalog of functions associated with rhizosphere colonization and growth rate potential

**Fig. 4:**
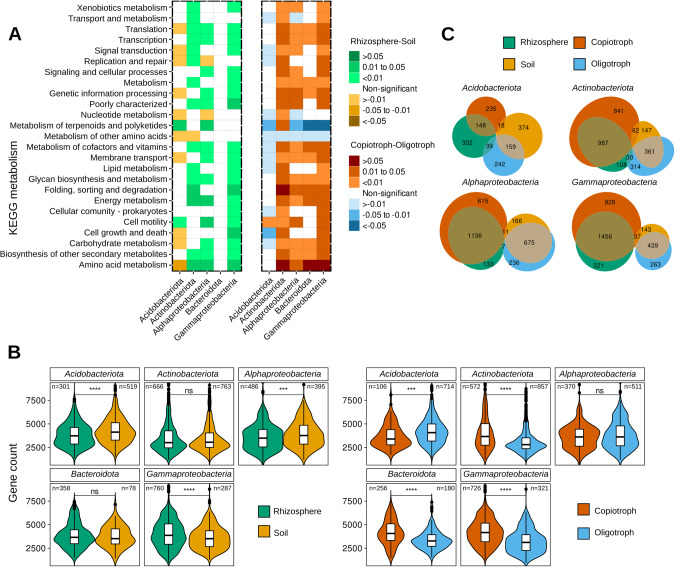
Comparison of metagenome-assembled genomes (MAGs) from rhizosphere/soils and fast-/slow-growers. **A** Enrichment of KEGG functional categories in MAGs from 5 most representative taxa. Differences in KO categories between rhizosphere and soil MAGs (left), and between copiotroph and oligotroph MAGs (right). Heatmaps indicate the level of enrichment based on the PhyloGLM test (adjusted *p* < 0.05, Benjamini-Hochberg FDR method). **B** Gene Counts in MAGs from rhizosphere or soil (left), and copiotroph or oligotroph (right). Number of MAGs in each category is indicated, distributions of predicted minimal doubling times in MAGs from rhizosphere and soils were compared with Mann–Whitney test (ns: *p* > 0.05, *p < =0.05, **p < =0.01, ***p < =0.001, ****p < =0.0001). **C** Euler plots with significantly enriched KO functions in MAGs. Plots show the number of enriched functions in the rhizosphere or soil, and copiotroph or oligotroph. Many more enriched functions were shared between rhizosphere-copiotroph and soil-oligotroph than between rhizosphere-oligotroph and soil-copiotroph.

To analyze which functions are significantly enriched when comparing MAGs from rhizospheres or soils and with growth rate status, we employed a phylogenetic-aware approach (PhyloGLM) to compare genome functional content (KEGG orthology, KO). We observed that in MAGs affiliated with *Actinobacteria*, *Alphaproteobacteria*, *Bacteroidota*, and *Gammaproteobacteria*, most functional categories were enriched in copiotrophs, i.e. 12 out of 25 categories in these four phyla, and 17 in at least 3 of these phyla. In contrast, significantly enriched functional categories in *Acidobacteria* were mostly associated with oligotrophs (Fig. 4A). This highlights the differences in functional categories present in the genomes of fast- and slow-growing bacteria in these taxa. We then compared the genome size between the groups (estimated as gene counts per genome, Fig. 4B) and found significantly larger genomes in copiotroph MAGs affiliated with *Actinobacteria*, *Bacteroidetes*, and *Gammaproteobacteria*, and in oligotrophic MAGs affiliated with *Acidobacteria*, while no difference in genome sizes was found in MAGs affiliated with *Alphaproteobacteria*, consistent with the enrichment of different functions in copiotrophs and in oligotrophs (Fig. 4A). Despite this difference in genome content, oligotrophs showed consistent enrichment in metabolism of terpenoids and polyketides, and metabolism of other amino acids, which include functions that are potentially relevant to the oligotrophic lifestyle. A similar pattern of genome content variation can be observed when comparing enriched processes in rhizosphere or soils in MAGs affiliated with *Acidobacteria* and *Gammaproteobacteria*, although no significant differences in genome content were found in MAGs affiliated with *Bacteroidetes* and *Actinobacteria*, and smaller genomes were found in MAGs affiliated with *Alphaproteobacteria* in rhizospheres, compared to those from soils. These patterns were also consistent with the enrichment of the different metabolisms in MAGs from rhizospheres or soils. Investigating why these differences in genome size exist in each taxonomic group and which functions are frequently missing in the smaller genomes could improve our understanding of copiotrophic and oligotrophic lifestyles.

When testing the enrichment of individual KO, COG, and Pfam functions, we observed that many enriched functions overlap between rhizosphere-enriched bacteria and copiotrophs, and between soil-enriched bacteria and oligotrophs (Fig. 4C, Supplementary Figs. 8–11, Supplementary Table 6). A higher number of significantly enriched functions were obtained in most represented taxa, especially in MAGs affiliated with *Alphaproteobacteria*, *Gammaproteobacteria*, *Acidobacteria*, and *Actinobacteria*.

### Growth rate potential is the main predictor of rhizosphere enrichment

To assess which functional features were important for copiotrophs or oligotrophs, we trained Random Forest (RF) and Gradient Boosting Classifier (GBC) models to predict the rhizosphere- or soil-association of a MAG based on a binary matrix including the presence or absence of KOs, COGs, and Pfams, as well as its status as a copiotroph or oligotroph. We used Grid Search with Stratified Cross-Validation to evaluate how changing different parameters affected the RF and GBC models (Supplementary Fig. 13–15, Supplementary Table 7). Using optimal parameters, we obtained F1-scores of 93.1, 92.5, and 92.6% (RF) and 93.8, 93.8, and 93.5% (GBC) for KO, COG, and Pfam-based models, respectively.

We used the classifiers to identify which features were associated with the rhizosphere or soil MAGs. All six models (i.e. RF/GBC models based on KO, COG, and Pfam) identified PMDT as the most important feature, suggesting that growth rate potential is highly relevant for rhizosphere colonization (Supplementary Fig. 16–17). We selected 5 h as a threshold to binarize based on a sensitivity analysis for this binarized variable using different thresholds (PMDT using 0.5,1,1.5,2,3,4,5,6, and 7 h, see Supplementary Table 8). We show that PMDT is the most important feature in both COG-, KO-, and Pfam-based RF models when using 5 h as threshold, coinciding with the threshold suggested previously [23]. The predictive importance of PMDT decreases when using other cutoffs values, although it always ranks within the top-10 (Supplementary Table 8). Growth rate potential is a complex microbial trait, estimated here from codon usage patterns. It may be associated with some of the functional features that are also used as predictors in the models. However, our results clearly show that this trait is more important than any other individual function, highlighting the high predictive potential of this complex microbial trait that is readily inferred from the genome sequence.

We further compared growth rate potential with other complex genomic traits, such as different metabolic modules composed of many individual functions. Thus, we used PhyloGLM models based on the presence or absence of 445 functional modules, as well as the PMDT to identify which individual modules are associated with rhizosphere colonization (Supplementary Table 9, Supplementary Fig. 12). We also calculated RF models using binary matrices composed of these composite features, revealing that even when compared with aggregated features of collective functions that may also be important for rhizosphere competence, such as flagellar assembly, PMDT is the most important feature for differentiating soil- and rhizosphere-associated bacteria (Supplementary Fig. 18). However, the overall performance of these models is lower than those using individual functions as features. Thus, despite being a complex trait based on codon usage bias, PMDT is more relevant for predicting rhizosphere colonization than any of the other composite traits tested here, including relevant metabolic pathways, hydrolytic enzymes, or motility.

### Niche and growth rate potential associated functional traits

**Fig. 5:**
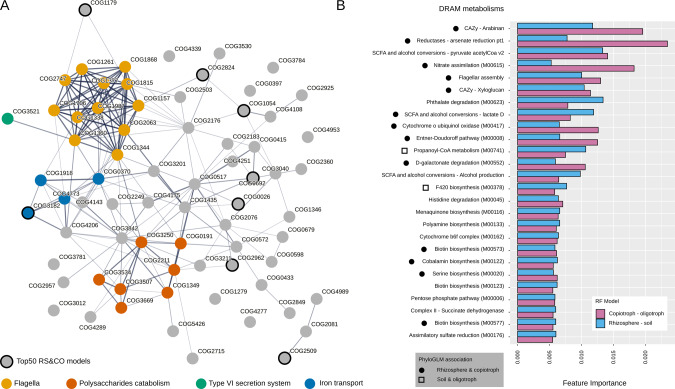
Analysis of strongly rhizosphere and growth-rate associated functions. **A** A STRING search of the COGs that were most important for predicting rhizosphere-association in the RF models, and were enriched only in the rhizosphere and in fast growing MAGs in at least one phylum according to the PhyloGLM analysis. Top 200 most important COGs in the RF models were sorted decreasingly by feature importance, and only COGs that were associated with both rhizosphere and fast-growing MAGs and never with soil or slow growing MAGs in the PhyloGLM models were selected, resulting in 71 COGs uniquely associated with the rhizosphere and fast-growing MAGs. Edge weights represent the level of evidence for functional interaction according to STRING. Some relevant functions are colored according to legend. Common COGs among the top 50 important features in RF models to predict niche and growth rate status were labeled as “Top50 RS&CO models”. **B** Top 50 most important modules in DRAM-based RF models to predict niche and growth rate status were selected and only common features are represented in a barplot. Black circles represent modules that are also uniquely associated with rhizosphere and copiotrophs, while squares represent those only associated with oligotrophs and soil in the PhyloGLM results.

Besides growth rate potential, the machine learning models trained above also enabled us to rank the functional features by their importance in classifying rhizosphere or soil bacteria (Supplementary Table 10). To visualize the functions that are associated with fast-growing bacteria in the rhizosphere, we selected the most important COGs that were exclusively associated with rhizosphere and copiotrophs based on the PhyloGLM results (Supplementary Table 6). We observed that many of these COGs were functionally connected in the STRING database [42] (Fig. 5A). One cluster comprises functions related to flagellar motility, which has been identified as an important trait for rhizosphere colonization in previous studies [55, 56], as it allows fast-growing bacteria to reach valuable exudate-derived carbon faster than the non-motile oligotrophs, and thus outcompete them in the rhizosphere. Linked to this cluster, we found an inter-membrane structural component of the type VI protein secretion system (T6SS, COG3521), which has been associated with modulating microbial interactions and promoting rhizosphere colonization by plant-beneficial bacteria [57, 58]. Other COGs belonging to T6SS were also associated with either the rhizosphere or copiotrophs, especially in MAGs affiliated with *Gammaproteobacteria* (Supplementary Table 6). Another connected cluster of COGs consists of proteins related to sugar catabolism, such as beta-galactosidase (COG3250), alpha-L-fucosidase (COG3669), alpha-L-arabinofuranosidase (COG3534), beta-xylosidase (COG3507), a Na + /melibiose symporter (COG2211), a DNA-binding transcriptional regulator of sugar metabolism of DeoR/GlpR family (COG1349), and a fructose/tagatose bisphosphate aldolase (COG0191), whose functions are implicated in mucilage polysaccharide degradation [59]. In a recent study in which the adaptation to plant colonization was tested in *Bacillus thuringiensis*, the authors found that metabolic pathways related to plant polysaccharides were upregulated in the adapted strain, including metabolism of various carbohydrates, such as cellobiose and galactose [60]. Supporting this observation, transporters of different sugar or sugar-derived molecules such as melibiose (K10117, K10118, K10119), glucarate (K03535), inositol (K06610, K06609), hexuronate (K08191), D-galactonate (K08194), maltose (K16211), gluconate (K06155) and other (K03291, K03328, K08139, K10111, K10546, K10547, K19270) were also exclusively enriched in rhizosphere and fast growers (Supplementary Table 6). Another cluster is related to iron uptake and may indicate the importance of rhizospheric fast-growing bacteria in maintaining iron homeostasis. This trait has recently been shown in the rhizosphere under drought conditions [61]. Moreover, we analyzed which of these features were also among the most important features in an RF to predict growth rate status using COGs (Supplementary Table 11, Fig. 5A). Among the top most important common features in both models, we found a tRNA U34 5’-hydroxylase (COG1054) and a tRNA A37 threonylcarbamoyladenosine dehydratase (COG1179), which expand decoding capabilities by modifying tRNA molecules in bacteria [62, 63]. We hypothesize that these modifications facilitate growth by improving codon-anticodon recognition in highly expressed and highly conserved genes [64]. We also found an uracil-DNA glycosylase (COG0692), which initiates the base-excision repair pathway and may indicate different uracil repair strategies in copiotrophs and oligotrophs [65, 66], a putative translation regulator (COG1179), an iron-regulated membrane protein (COG3182), and an enzyme involved in purine biosynthesis (COG0026), among other less-characterized COGs (COG2824, COG2962, COG2509; Fig. 5A). All these functions probably facilitate high growth rates which also allows a successful rhizosphere colonization.

To gain a broader insight, we analyzed the common functional modules that were most important in predicting the niche and growth rate status in separate DRAM-based RF models (Fig. 5B, Supplementary Table 11). We only found propanoyl-CoA metabolism (M00741), and cofactor F420 biosynthesis (M00378) to be uniquely associated with soil and slow-growers in PhyloGLM results (Supplementary Table 9). These functions are probably involved in utilizing recalcitrant carbon sources present in the soil [67, 68]. On the other hand, we found more modules associated with rhizosphere and fast-growing MAGs, notably CAZy enzymes related to the utilization of arabinan and xyloglucan. Arabinan has been shown to be upregulated in *Escherichia coli* O157:H7 colonizing different plants [69], and xyloglucans are recalcitrant polysaccharides that compose plant cell walls and are depolymerized by fast growing plant pathogens and gut commensals [70]. In fact, we found other CAZy enzymes to degrade mixed-linkage glucans, polyphenolics, sulfated polysaccharides, and amorphous cellulose associated both with rhizospheric and fast-growing bacteria, but we did not find any CAZy enzymes significantly associated with soil or slow-growing bacteria (Supplementary Table 9). We also observed the flagellar assembly module to be only associated with the rhizospheric and fast-growing bacteria. Other modules associated with rhizosphere and fast-growing MAGs included D-galactonate degradation (M00552), which was recently described as a rhizosphere-specific determinant in *Pseudomonas putida* populations [71], nitrate assimilation (M00615), arsenate reduction, a trait frequently explored in rhizosphere bacteria with bioremediation purposes [72], cobalamin biosynthesis, a trait present in a small proportion of soil bacteria [73], and different biotin biosynthesis pathways (Fig. 5B).

### Ecological insight

Overall, we hypothesize that rhizosphere-associated bacteria might profit from nutrients coming from root exudates, using expensive flagellar motility and a huge diversity of transporters and enzymes to reach, internalize, and catabolize these compounds, altogether allowing them to achieve faster growth rates. To grow faster, these bacteria have optimized the codon usage of their ribosomal proteins, and improved their decoding capabilities and protection of their DNA from mutagenesis. Our work complements recent evidence showing generalized effects on rhizosphere microbiomes. A recent study analyzed 557 paired bulk soil/rhizosphere datasets using a network approach to identify rhizosphere-associated signatures [74]. They found sporulation enriched in bulk soils, while carbon and nitrogen transformation, methanol oxidation, and methylotrophy were enriched in the rhizosphere. The authors also showed that rhizospheric bacteria had 6.6% more rRNA operons than soil bacteria. Because copiotrophs (r-strategists) are assumed to have more rRNA operons than oligotrophs (K-strategists) [75], this analysis also indicated that fast-growing bacteria preferentially colonize the rhizosphere. Another recent study used stable isotope probing, quantitative PCR, marker gene sequencing, and shotgun metagenomic sequencing to examine rhizosphere taxa that incorporated plant-derived 13 C [76]. While the bacteria that consumed plant-derived carbon were not necessarily the most abundant in the rhizosphere, they had higher estimated growth rates and encoded genes associated with carbon metabolism, resource uptake, and the potential for promoting plant growth.

We found more rhizosphere- and soil-associated functions than a previous study by Levy et al. [14], probably because we included a larger set of genomes including both culturable and unculturable bacteria, but also spanning a broader taxonomic range. Approximately 66% of Levy’s significant COGs overlapped with COGs associated with rhizosphere and soil in our analysis (Supplementary Fig. 19). Our STRING analysis further revealed that the COGs identified herein were functionally similar, suggesting that our analysis both complements and expands on the previous study by identifying hundreds of new COGs involved in rhizosphere colonization.

## Conclusions

Understanding which factors drive the rhizosphere effect is important for designing strategies to harness plant microbiomes and develop more resilient bio-inoculants. We demonstrated that rhizosphere-enriched bacteria have higher growth rate potential than those in soils. This observation holds in eleven of the most abundant phyla independently of host plant genotype, stress condition, soil type, light cycle, or life stage. We used machine learning to accurately classify MAGs from rhizospheres or soils, finding PMDT to be the most important feature for classification, even compared to other broad functional modules. Our analysis identified important features known to be associated with rhizosphere colonization or copiotrophic lifestyle, including flagella, sugar and polysaccharide degradation, transporters, tRNA modifying enzymes, as well as novel proteins that may be further explored. The fact that growth rate potential is the most important predictor explaining rhizosphere association is consistent with the notion that the nutritional gradients generated by plant root exudates provide a selective environment for a subset of copiotrophic bacteria from the vast microbial diversity present in soils. Moving forward, determining how much of the growth rate potential reflects realized growth rates in nature, and establishing whether copiotrophic lifestyle necessarily implies high maximal growth rates will require more careful experimentation. As we have shown here, bacterial growth rate potential is the most important genomic predictor to our knowledge for determining the ability to colonize the rhizosphere.

## Supplementary information

Supplementary Figures

Suplementary Table 1

Suplementary Table 2

Suplementary Table 3

Suplementary Table 4

Suplementary Table 5

Suplementary Table 6

Suplementary Table 7

Suplementary Table 8

Suplementary Table 9

Suplementary Table 10

Suplementary Table 11

## Data Availability

R scripts and Jupyter Notebooks for analysis, plotting, and tables, metagenomic and genomic data, and annotations are available in Zenodo: 10.5281/zenodo.7725928 All visualizations were done using ggplot2 [77], ggpubr [78], and InkSkape [79].
