# Supplementary material for: Growth rate is a dominant factor predicting the rhizosphere effect
Source: ISME J. 2023 Jun 15;17(9):1396–405. doi: 10.1038/s41396-023-01453-6 (PMC10432406; doi:10.1038/s41396-023-01453-6)
Supplement: Supplementary file 1 — Supplementary Figures [file 41396_2023_1453_MOESM1_ESM.pdf]

Supplementary Figures

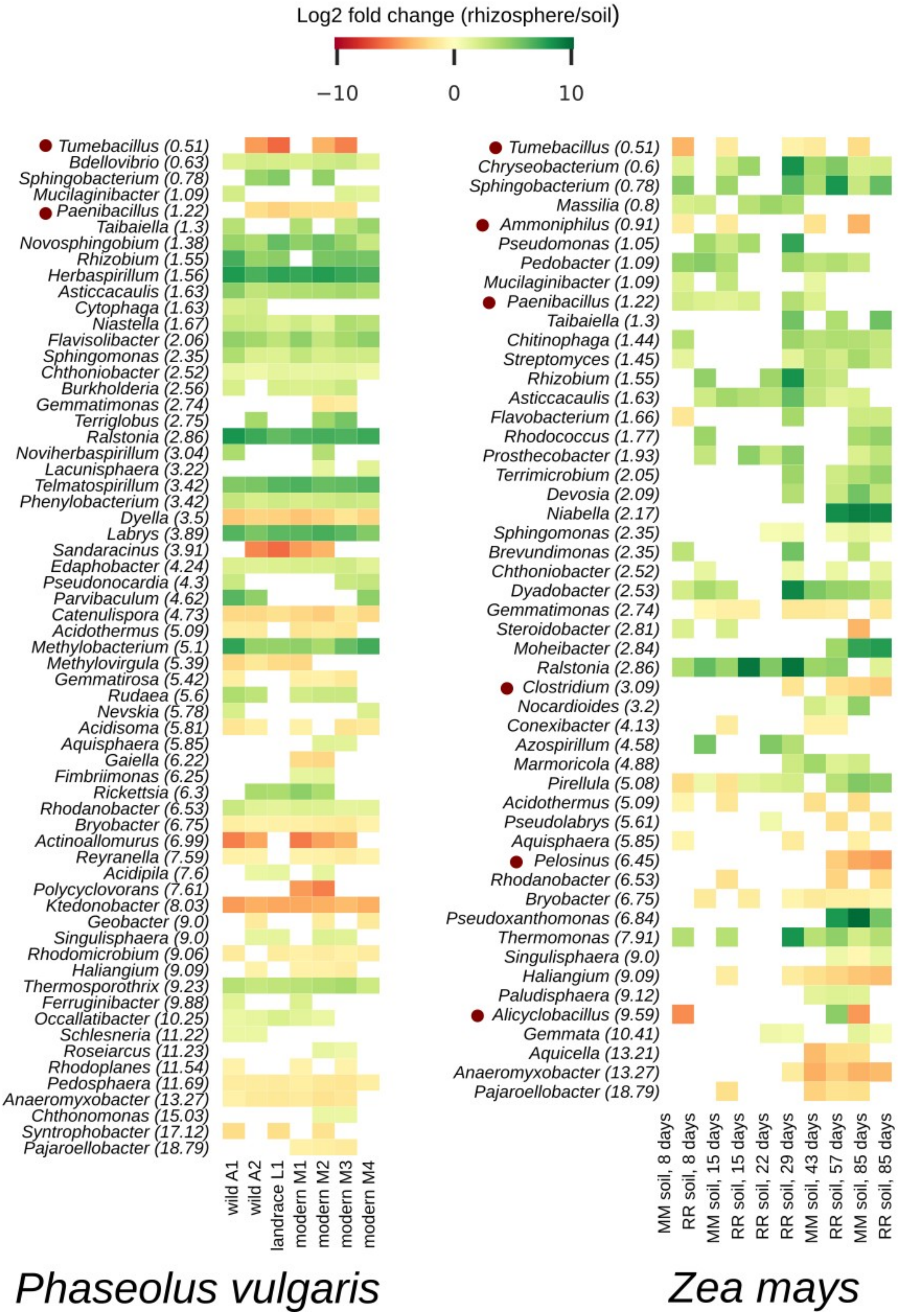

Supplementary Figure 1. Rhizosphere enrichment in wild and modern accessions of *Phaseolus vulgaris* (common bean), and in *Zea mays* grown in soils with different crop rotation systems. Differentially abundant genera between rhizosphere and bulk soil replicates were identified by DESeq2 analysis (p adj < 0.05), and shown here as being significant at least in one experiment. Genera are sorted from lower PMDT (copiotrophs, top) to higher PMDT (oligotrophs, bottom). Numbers in brackets indicate PMDT obtained from the EGGO database for each genus. Brown dots represent genera belonging to the *Firmicutes* phylum.

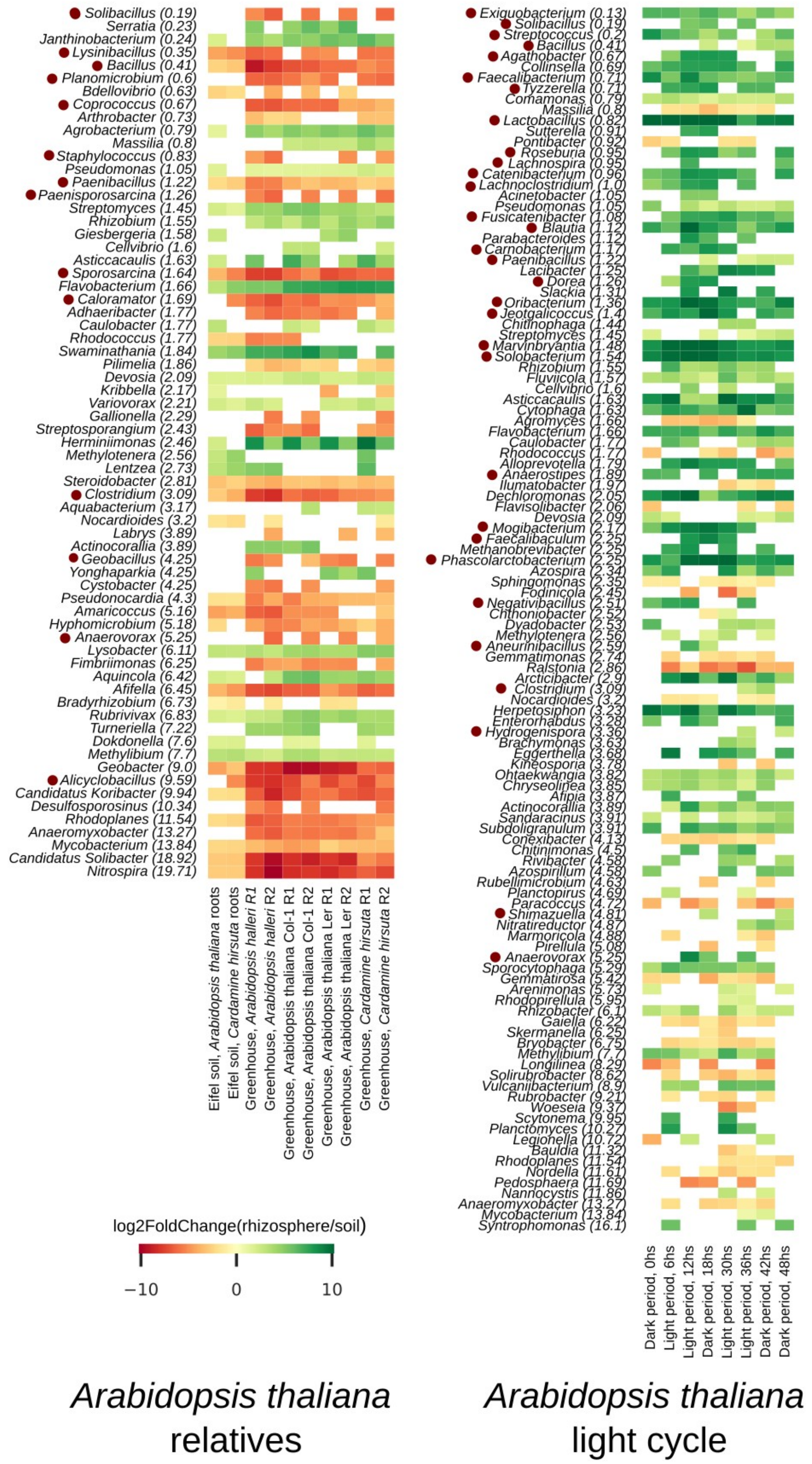

Supplementary Figure 2. Rhizosphere enrichment in *Arabidopsis thaliana* ecotypes and sister species, and *Arabidopsis thaliana* Col-0 ecotype under light-dark cycle. Differentially abundant genera between rhizosphere and bulk soil replicates were identified by DESeq2 analysis (p adj < 0.05), and shown here as being significant at least in one experiment. Genera are sorted from lower PMDT (copiotrophs, top) to higher PMDT (oligotrophs, bottom). Numbers in brackets indicate PMDT obtained from the EGGO database for each genus. Brown dots represent genera belonging to the *Firmicutes* phylum.

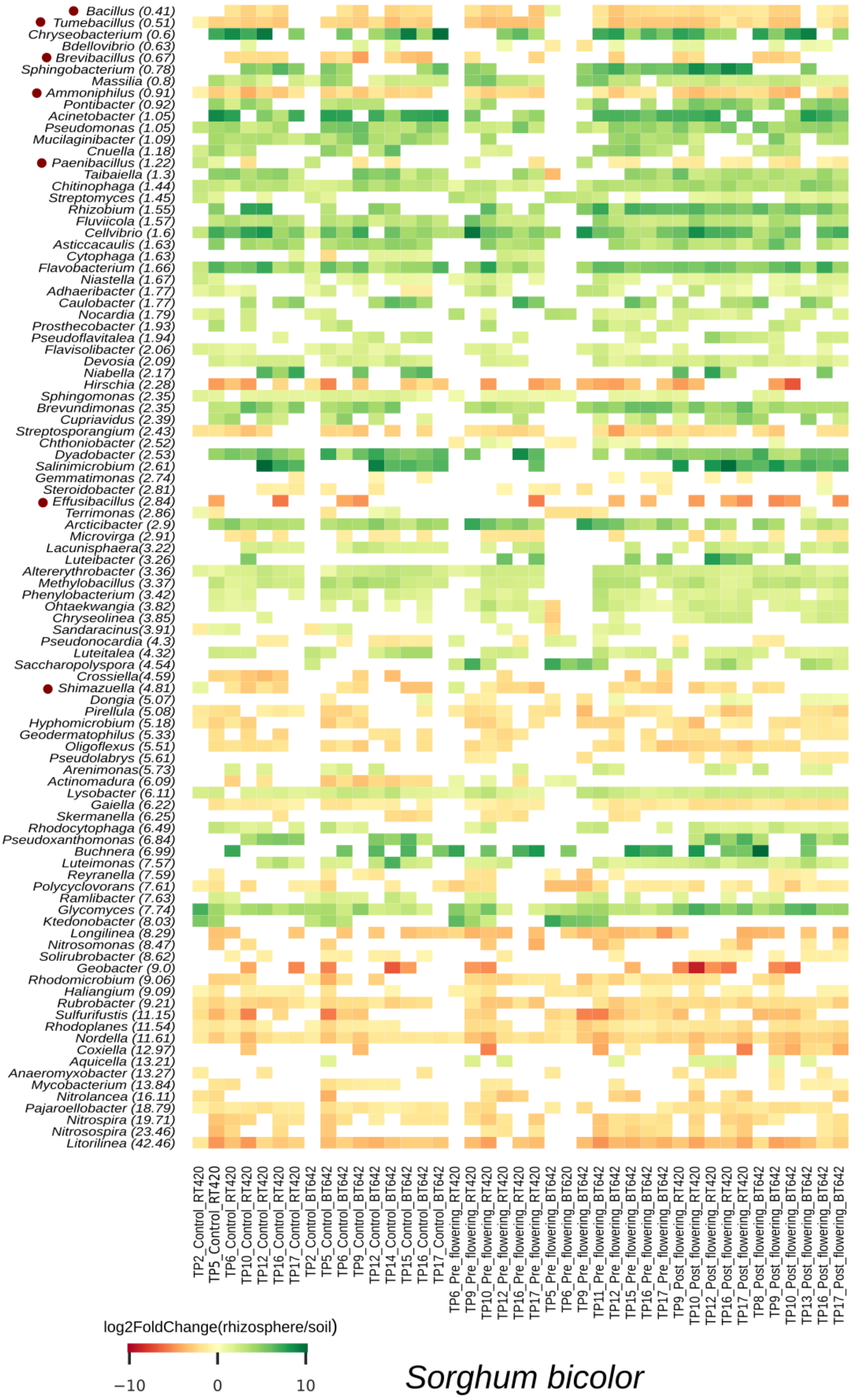

Supplementary Figure 3. Rhizosphere enrichment in *Sorghum bicolor* under drought stress and control conditions, at different timepoints in its lifecycle. Differentially abundant genera between rhizosphere and bulk soil replicates were identified by DESeq2 analysis (p adj < 0.05), and shown here as being significant at least in one experiment. Genera are sorted from lower PMDT (copiotrophs, top) to higher PMDT (oligotrophs, bottom). Numbers in brackets indicate PMDT obtained from the EGGO database for each genus. Brown dots represent genera belonging to the *Firmicutes* phylum.

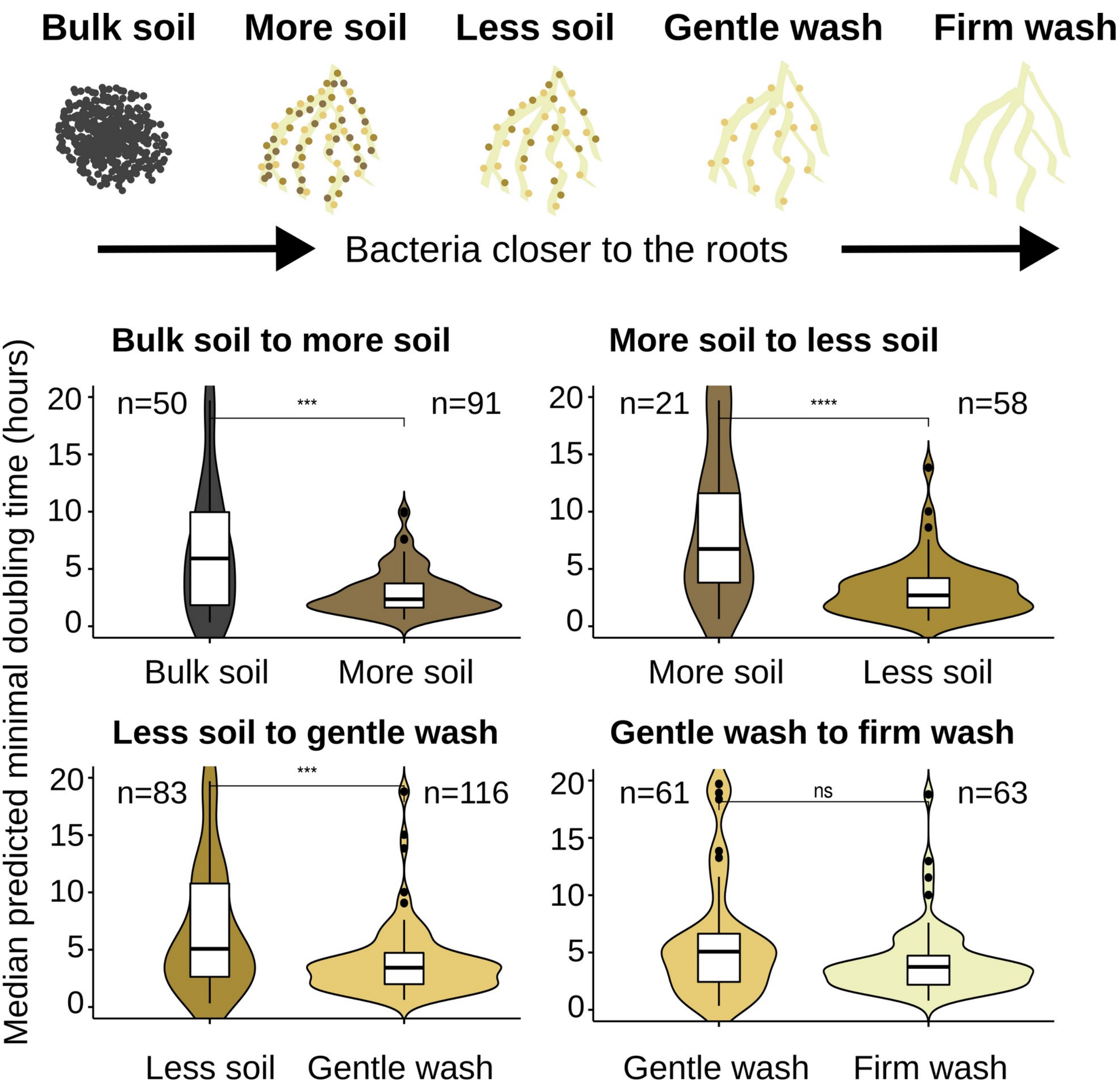

Supplementary Figure 4. PMDT distributions of genera enriched in *Arabidopsis thaliana* Col-0 sampled at different stages along a bulk soil-to-rhizosphere gradient. Differentially abundant genera between each pair of the different compartments in the bulk soil-to-rhizosphere gradient were identified by DESeq2 analysis (*p* adj < 0.05). PMDT of genera enriched in each compartment are represented. Stepwise compartments from bulk soil to rhizosphere were labeled based on the experimental design in Poppeliers et. al [45]. BS: bulk soil, MS: more soil, LS: less soil, GW: gentle wash, FW: firm wash. Distributions of predicted minimal doubling times in genomes from rhizosphere and soils were compared with Mann-Whitney test (ns: $p > 0.05$, *: $p <= 0.05$, **: $p <= 0.01$, ***: $p <= 0.001$, ****: $p <= 0.0001$).

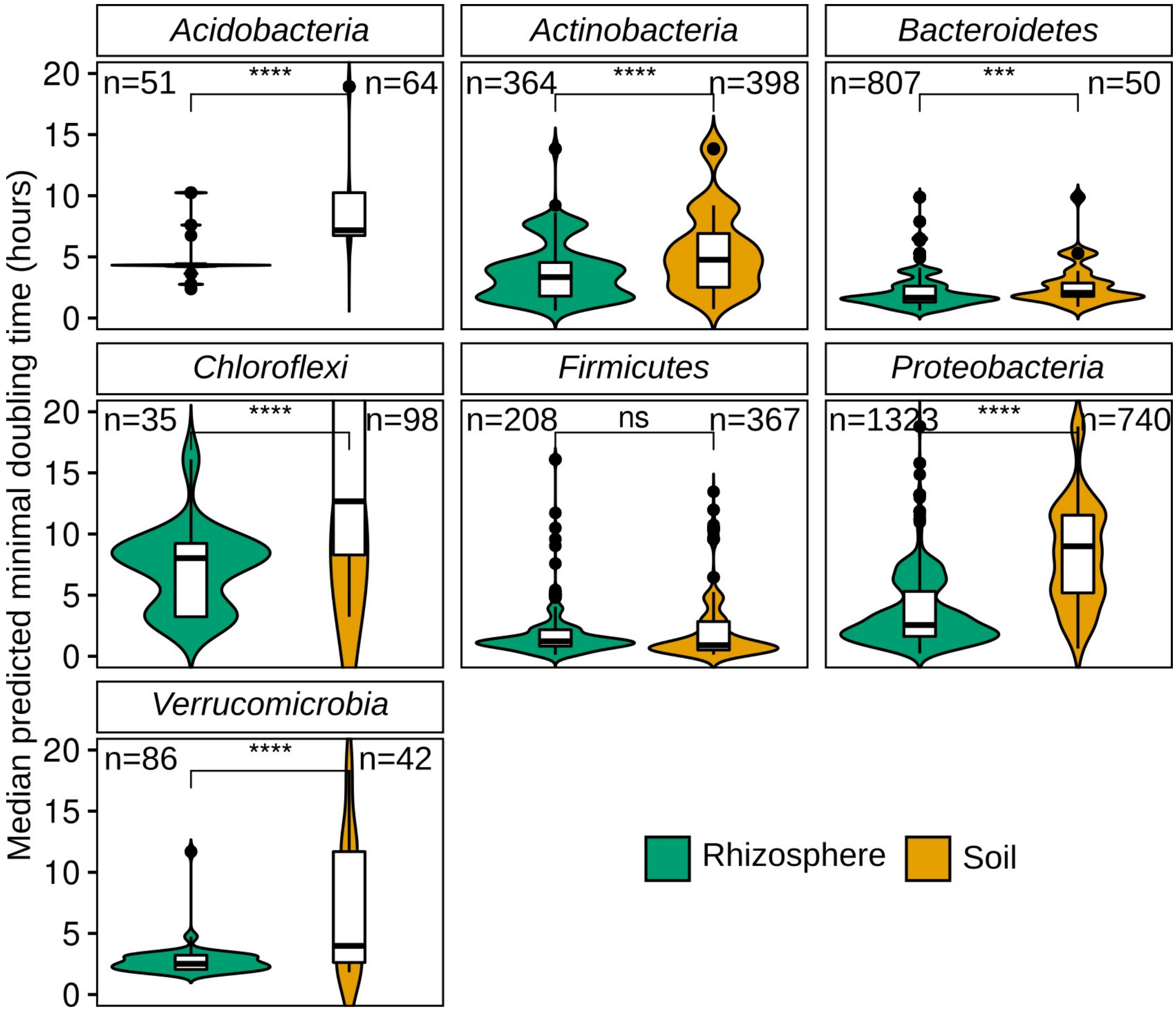

Supplementary Figure 5. PMDT in rhizosphere and soil enriched-genera by phylum. Significant enriched genera in all projects were merged and classified in phylum. Violin plots represent those belonging to the most representative phyla. Rhizosphere enriched genera present lower PMDT in *Acidobacteria*, *Actinobacteria*, *Bacteroidetes*, *Chloroflexi*, *Proteobacteria* and *Verrucomicrobia*, but not in *Firmicutes.* Distributions of predicted minimal doubling times in genomes from rhizosphere and soils were compared with Mann-Whitney test (ns: $p > 0.05$, *: $p <= 0.05$, **: $p <= 0.01$, ***: $p <= 0.001$, ****: $p <= 0.0001$).

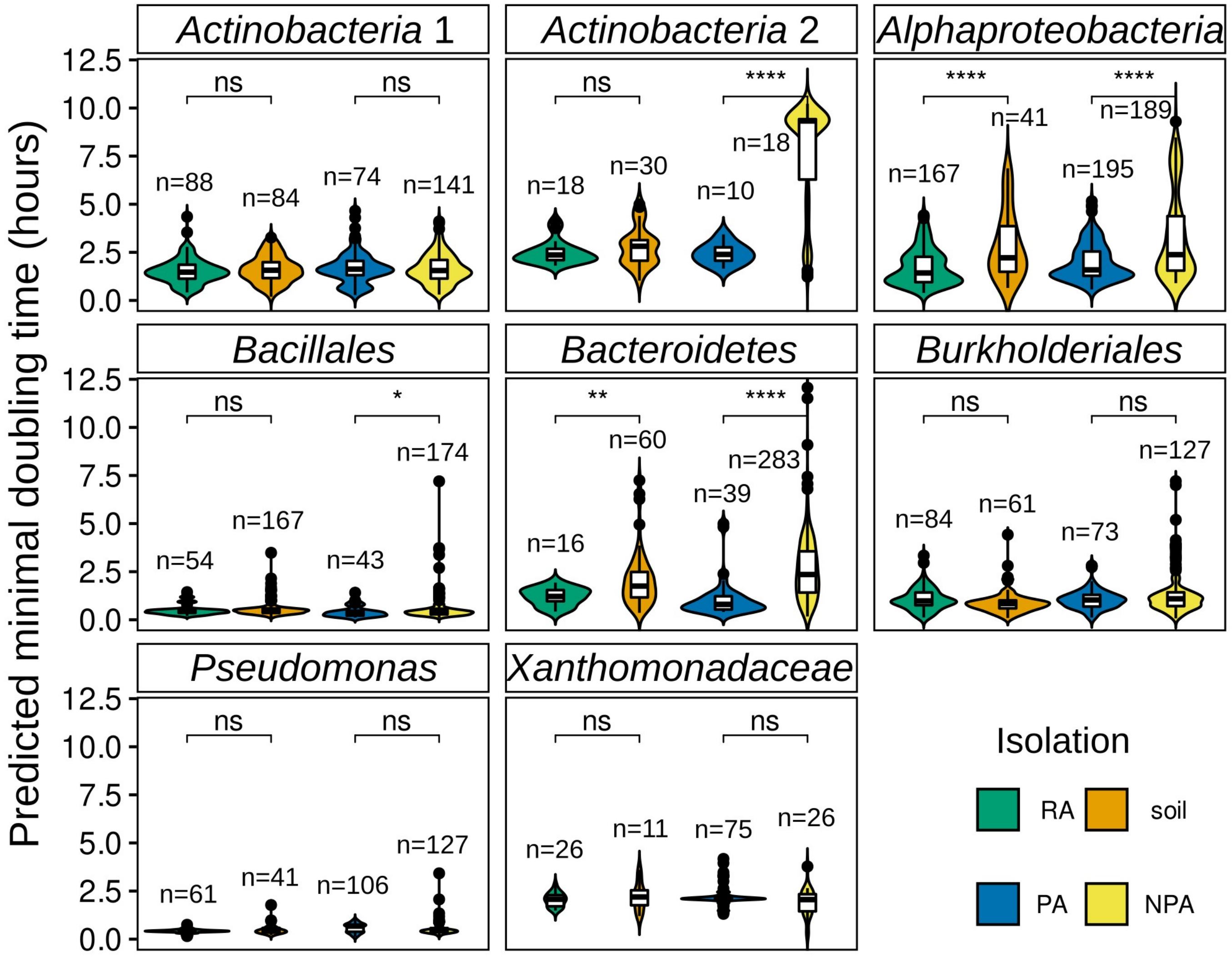

Supplementary Figure 6: PMDT in Levy et. al. culturable genomes. Significant differences between genomes isolated from rhizospheres and soils can only be observed in *Alphaproteobacteria* and *Bacteroidetes* groups. RA: root-associated environments (rhizoplane and endosphere); PA: plant-associated environments (including root-associated, and rhizosphere bacteria); NPA: non-plant-associated environments, including humans, non-human animals, air, sediments, and aquatic environments; soil: bacteria isolated from soils. Distributions of predicted minimal doubling times in genomes from rhizosphere and soils were compared with Mann-Whitney test (ns: $p > 0.05$, *: $p <= 0.05$, **: $p <= 0.01$, ***: $p <= 0.001$, ****: $p <= 0.0001$).

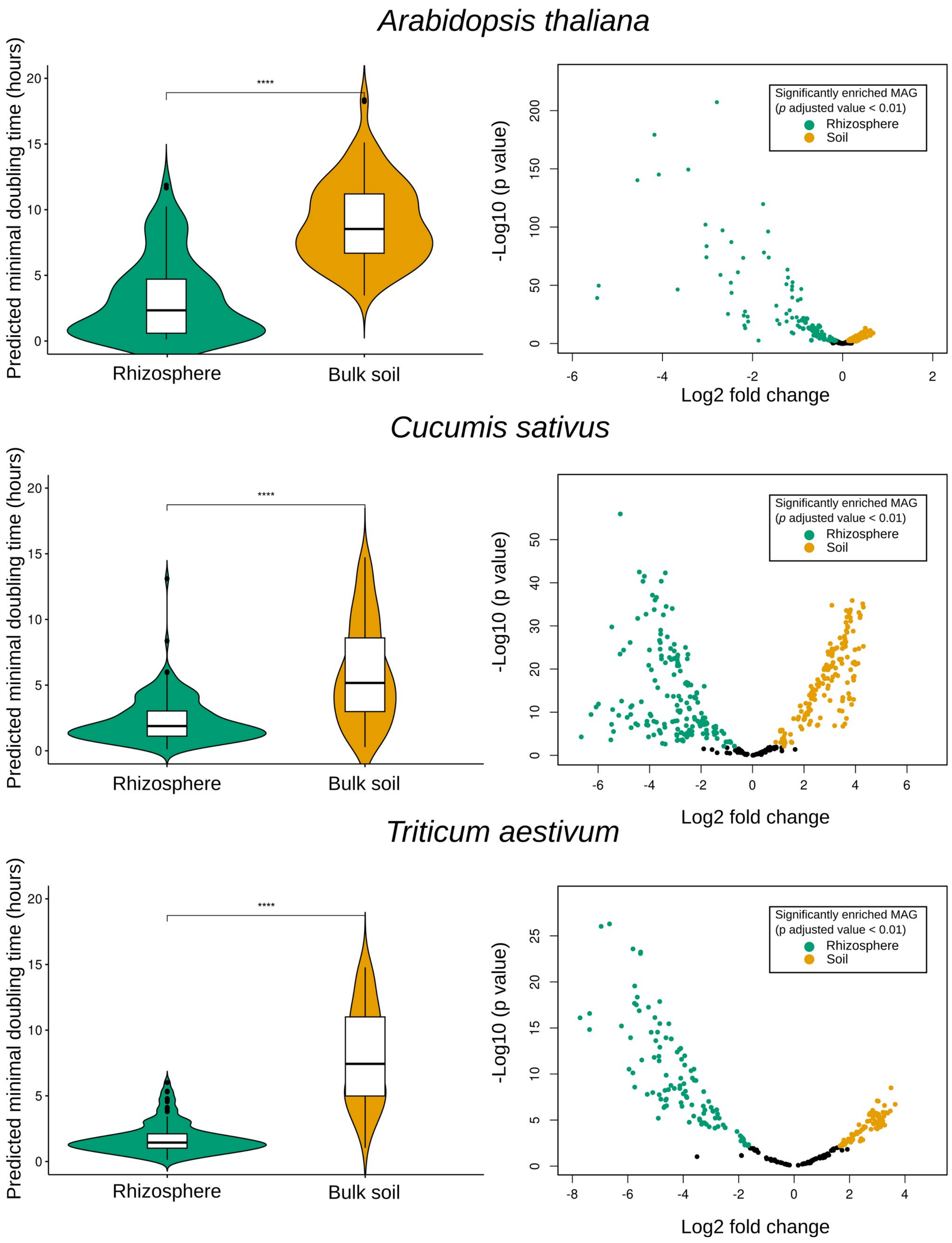

Supplementary Figure 7: PMDTs in MAGs enriched in rhizospheric and bulk soil samples. We mapped paired rhizophere and their bulk-soil whole metagenomic samples from *A. thaliana*, *C. sativus*, *and T. aestivum* to the catalog of MAGs obtained from diverse samples of rhizospheres and soils. We identified significantly enriched MAGs in rhizospheres or soils using DESeq2 analysis (p adj < 0.05). Then, PMDTs from MAGs enriched in the rhizosphere and in bulk soil were compared with Mann-Whitney test (ns: p > 0.05, *: p <= 0.05, **: p <= 0.01, ***: p <= 0.001, ****: p <= 0.0001).

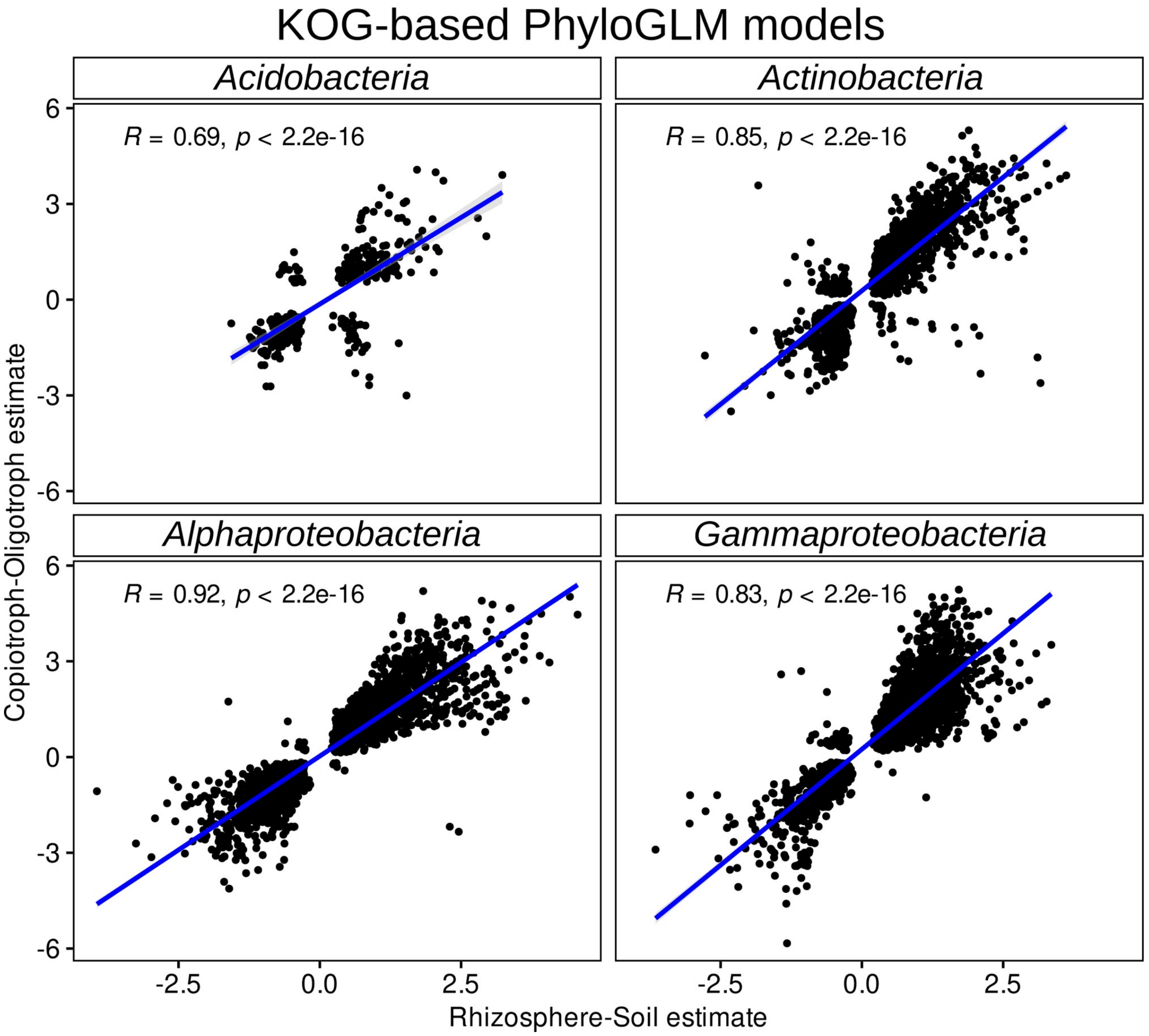

Supplementary Figure 8. KO-based PhyloGLM estimates correlation between rhizosphere-soil and copiotroph-oligotroph models. Significantly enriched KO functions in both models (Rhizosphere-Soil and Copiotroph-Oligotroph, PhyloGLM FDR < 0.05) positively correlate in *Alphaproteobacteria*, *Gammaproteobacteria*, *Acidobacteria*, and *Actinobacteria*, indicating that most functions enriched in rhizosphere MAGs are also enriched in copiotroph MAGs, and vice versa. Spearman correlation tests are shown.

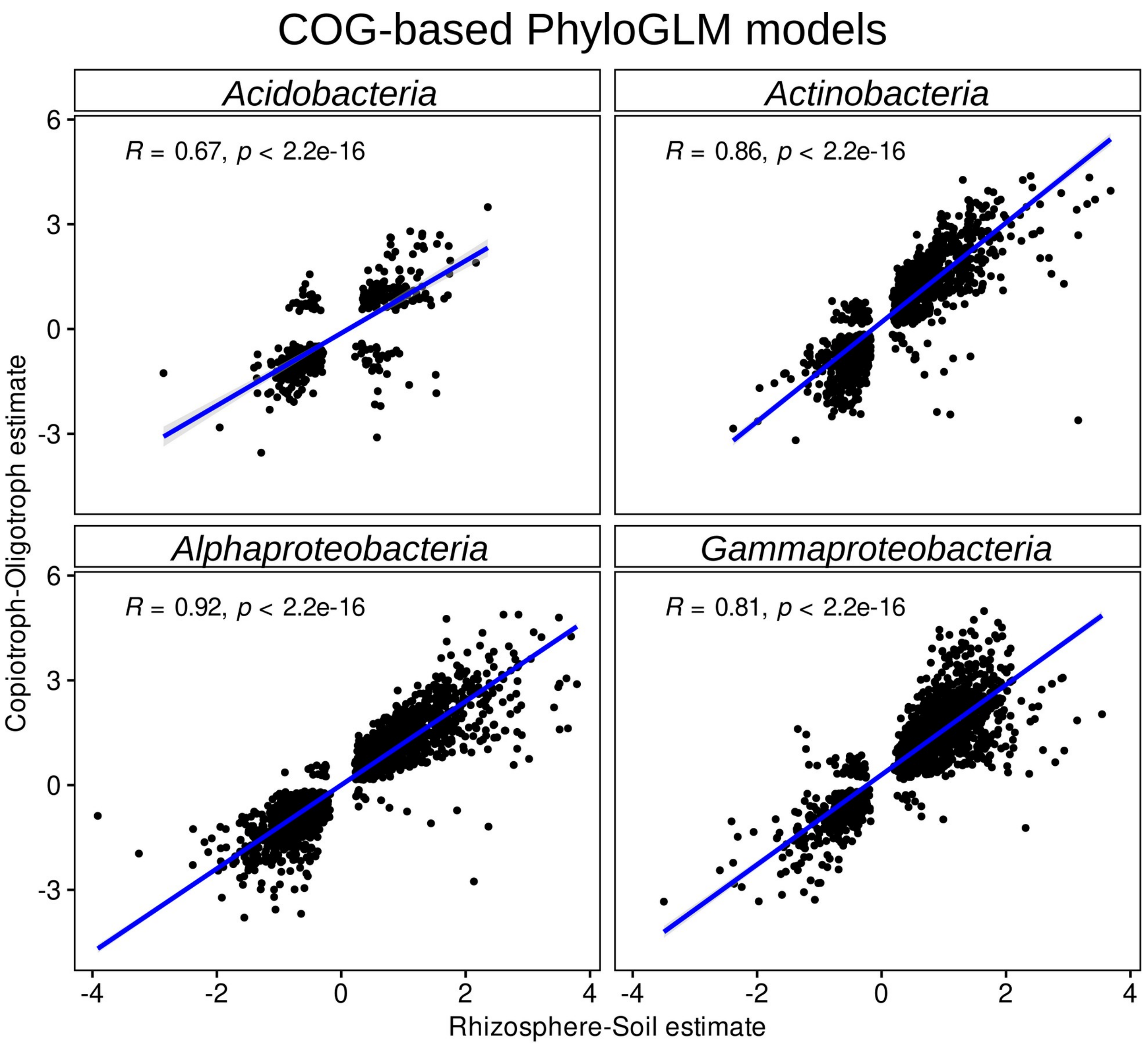

Supplementary Figure 9. COG-based PhyloGLM estimates correlation between rhizosphere-soil and copiotroph-oligotroph models. Significantly enriched COG functions in both models (Rhizo-Soil and Cop-Oli, PhyloGLM, FDR < 0.05) positively correlate in *Alphaproteobacteria*, *Gammaproteobacteria*, *Acidobacteria* and *Actinobacteria*, indicating that most functions enriched in rhizosphere MAGs are also enriched in copiotroph MAGs, and vice versa. *Bacteroidetes* show no correlation. Spearman correlation tests are shown.

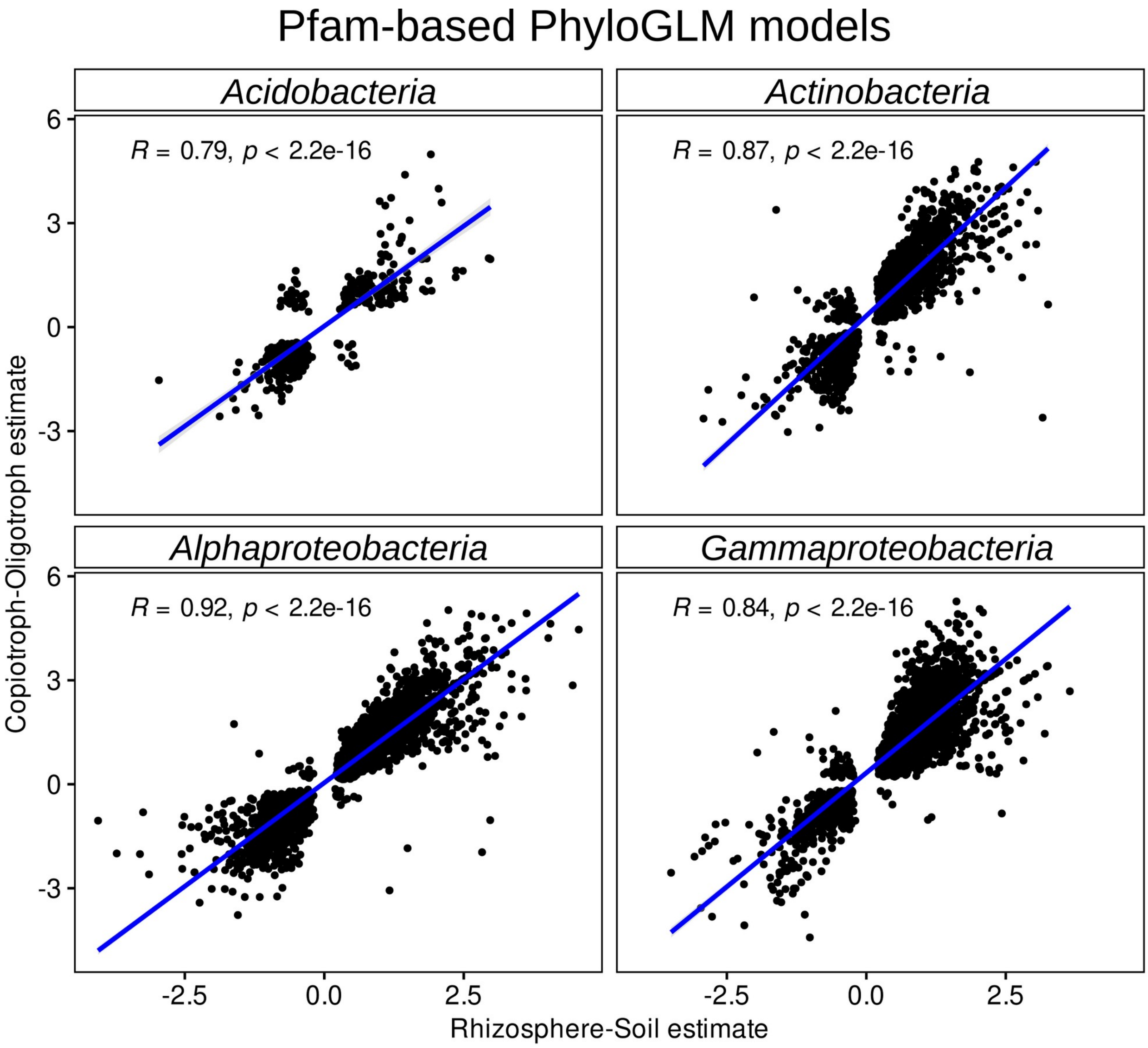

Supplementary Figure 10. Pfam-based PhyloGLM estimates correlation between rhizosphere-soil and copiotroph-oligotroph models. Significantly enriched Pfam functions in both models (Rhizo-Soil and Cop-Oli, PhyloGLM, FDR < 0.05) positively correlate in *Alphaproteobacteria*, *Gammaproteobacteria*, *Acidobacteria* and *Actinobacteria*, indicating that most functions enriched in rhizosphere MAGs are also enriched in copiotroph MAGs, and vice versa. Spearman correlation tests are shown.

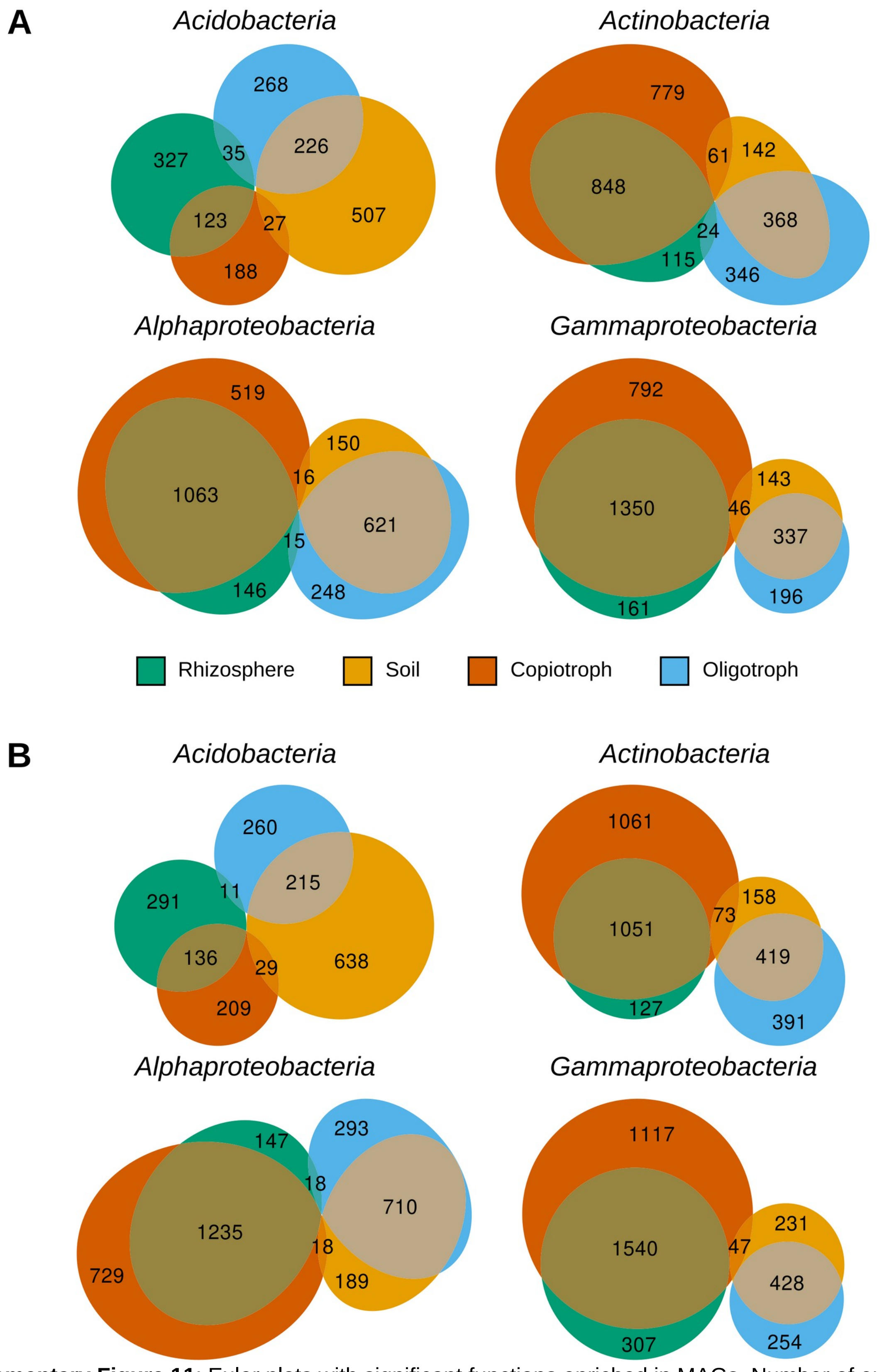

Supplementary Figure 11: Euler plots with significant functions enriched in MAGs. Number of enriched functions in PhyloGLM models in rhizospheres (green) or soil (brown), and in copiotrophs (red) or oligotrophs (blue). Functions mainly enriched in MAGs from rhizospheres are also the same being enriched in copiotrophs, similarly functions enriched in soils are mainly enriched in oligotrophs. A. COG-based PhyloGLM models. B. Pfam-based PhyloGLM models.

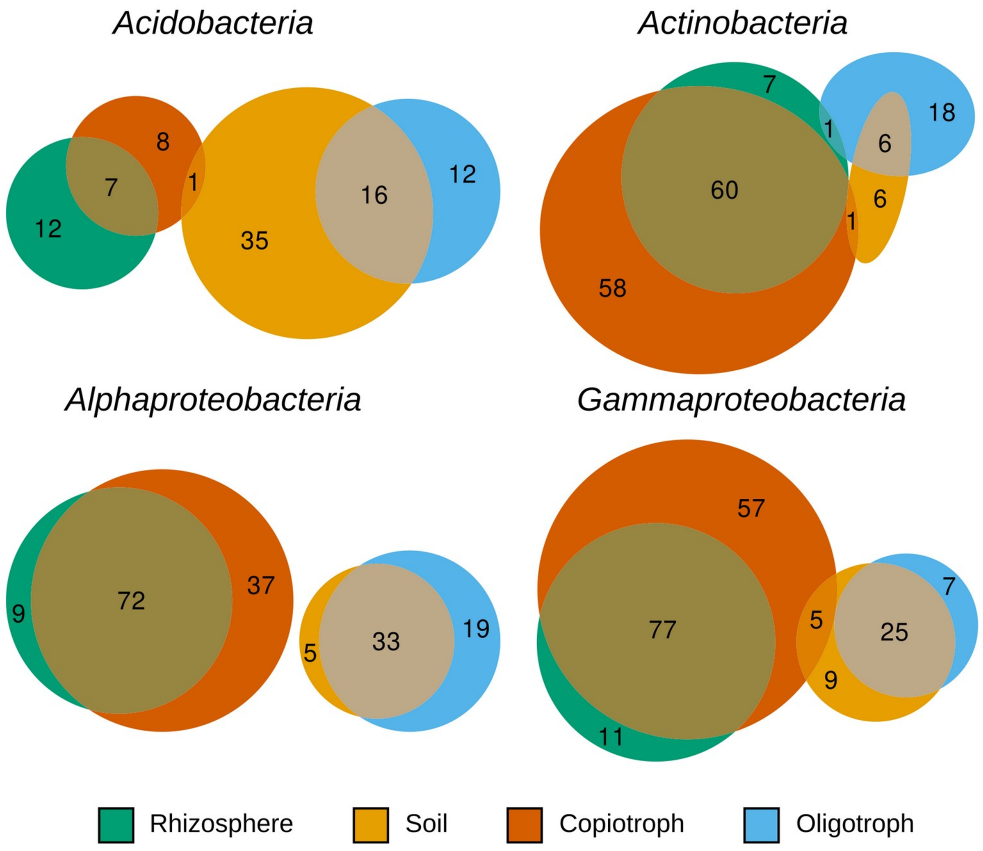

Supplementary Figure 12: Euler plots with functional modules enriched in MAGs. Functional modules were annotated using DRAM. Number of enriched modules in PhyloGLM models significantly associated to rhizospheres or soil biomes, or to copiotrophs or oligotrophs growth rate (FDR < 0.05). Modules mainly enriched in MAGs from rhizospheres are also the same being enriched in copiotrophs, similarly modules enriched in soils are mainly enriched in oligotrophs. The results showed an overlap in the functional modules associated with rhizosphere-enriched bacteria and copiotrophs, and between soil-enriched bacteria and oligotrophs, similar to what was observed with individual functions (Supplementary Figure 11).

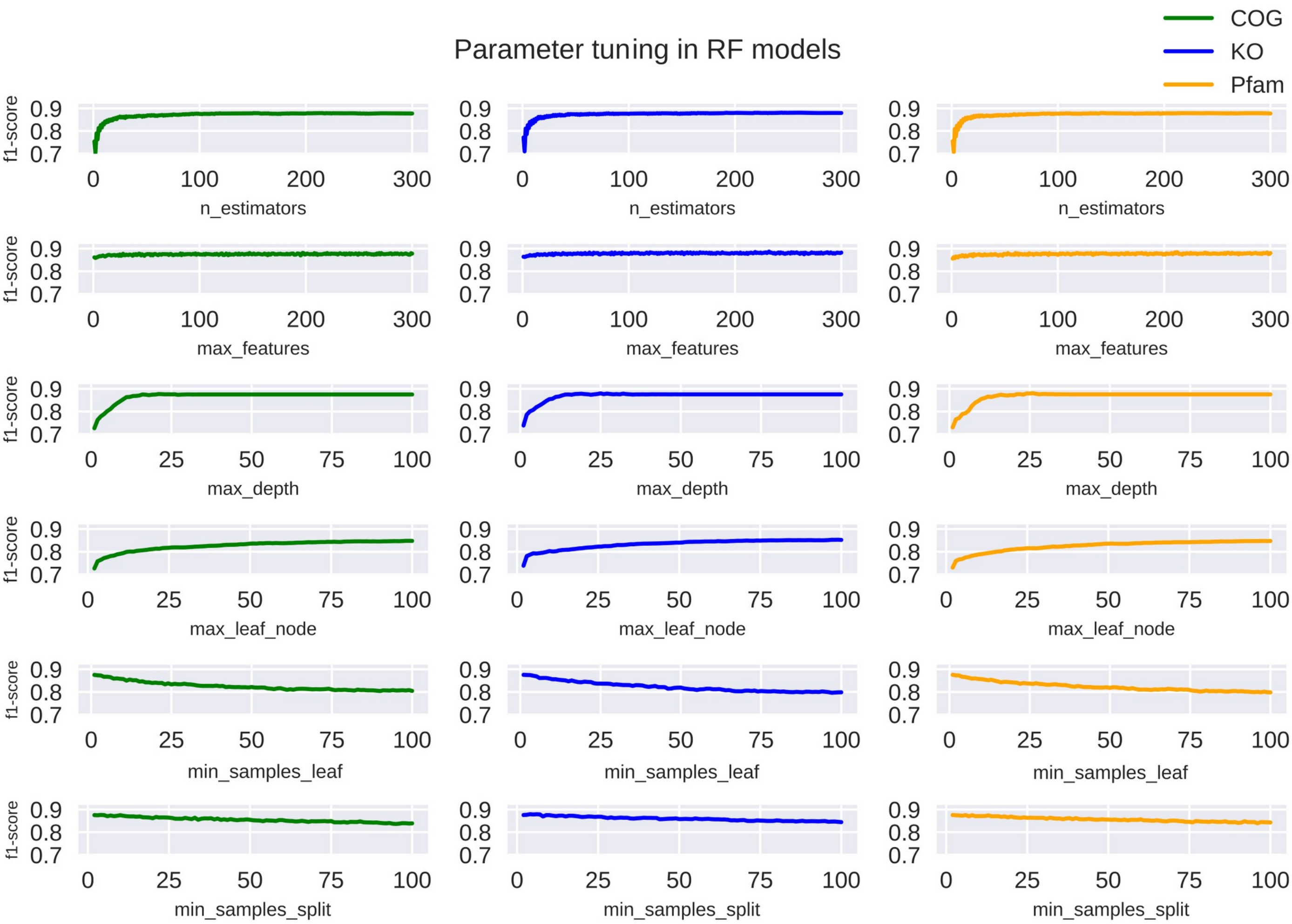

Supplementary Figure 13: Parameter tuning for Random Forest models. RF classification models were trained using COG, KO and Pfam binary matrices with copiotroph or oligotroph as an additional features. We observed that increasing the number of trees (n_estimators) above 60 did not significantly increase the F1-score with neither COG, nor KO, nor Pfam matrices. Also, changing the maximum number of features assessed at each node (max_features) or other pre-pruning parameters (see methods) did not result in a significant improvement of the RF models. Thus, we set the number of trees to 300 and used default values for the remaining parameters, obtaining overall 5-fold cross-validated accuracy scores of 92.3%, 91.6%, and 91.7%, precision scores of 92.1%, 91.2%, and 91.4%, recall scores of 94.1%, 93.8%, and 93.8%, and F1-scores of 93.1%, 92.5%, and 92.6% for KO, COG, and Pfam based models, respectively.

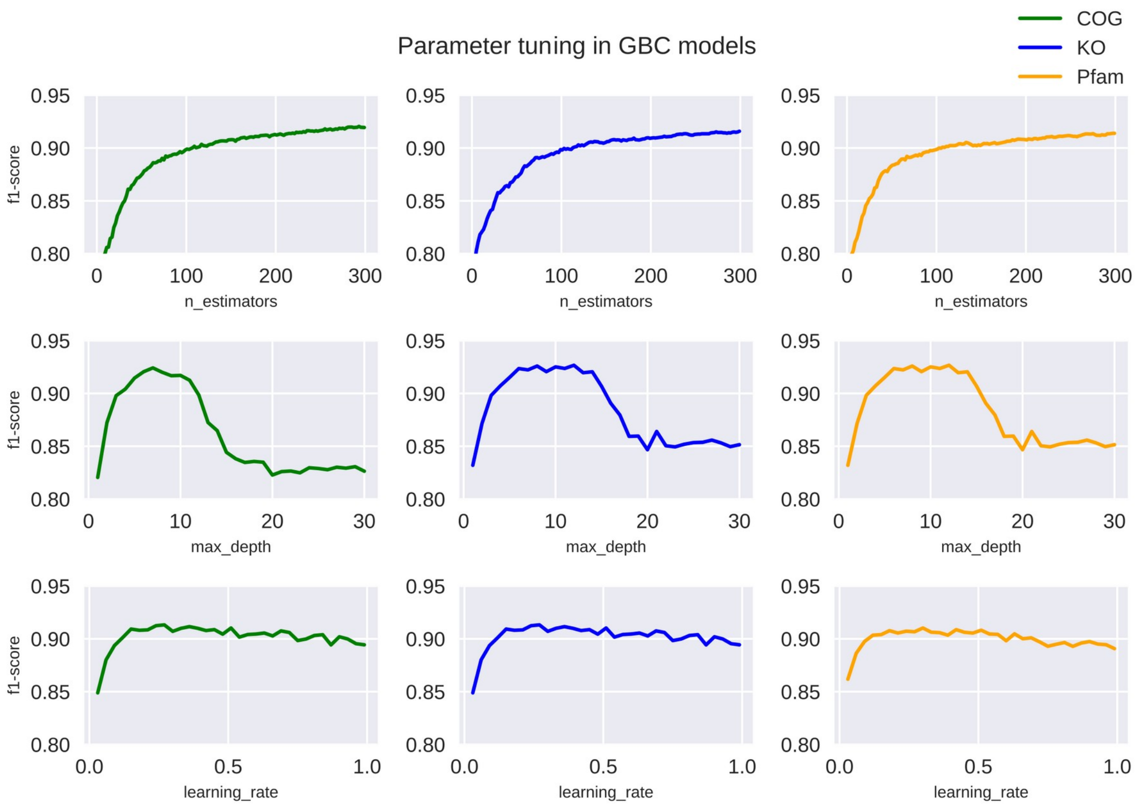

Supplementary Figure 14: Parameter tuning for Gradient Boosting Classifier models. Parameters n_estimators, max_depth, and learning_rate were changed, and f1-scores were used as criteria to define optimal ranges for these parameters.

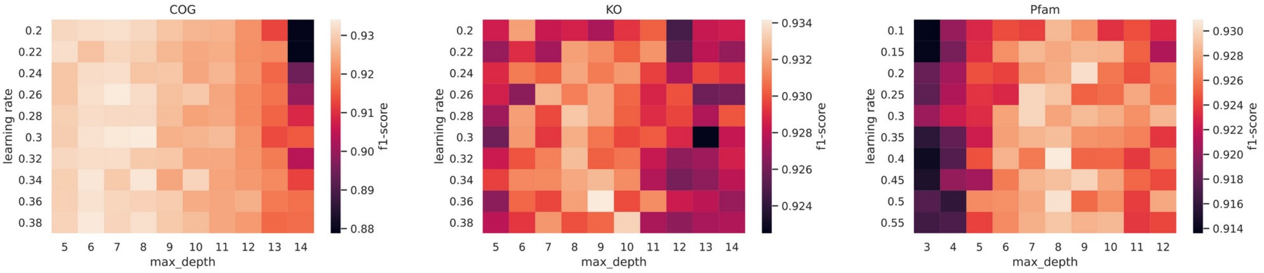

Supplementary Figure 15: F1-scores of a grid search combining max_depth and learning_rate parameters in GBC COG (A), KO (B) and Pfam (C) models, with n_estimators=300. Optimal parameters were max_depth=8, 9, and 8, and learning_rate=0.30, 0.36, and 0.40, respectively. With these parameters we obtained overall accuracy of 93.1%, 93.2%, and 92.8%, precision of 93.0%, 93.2%, and 92.6%, recall of 94.6%, 94.5%, and 94.4%, and F1-score of 93.8%, 93.8%, and 93.5% for KO, COG, and Pfam based models, respectively.

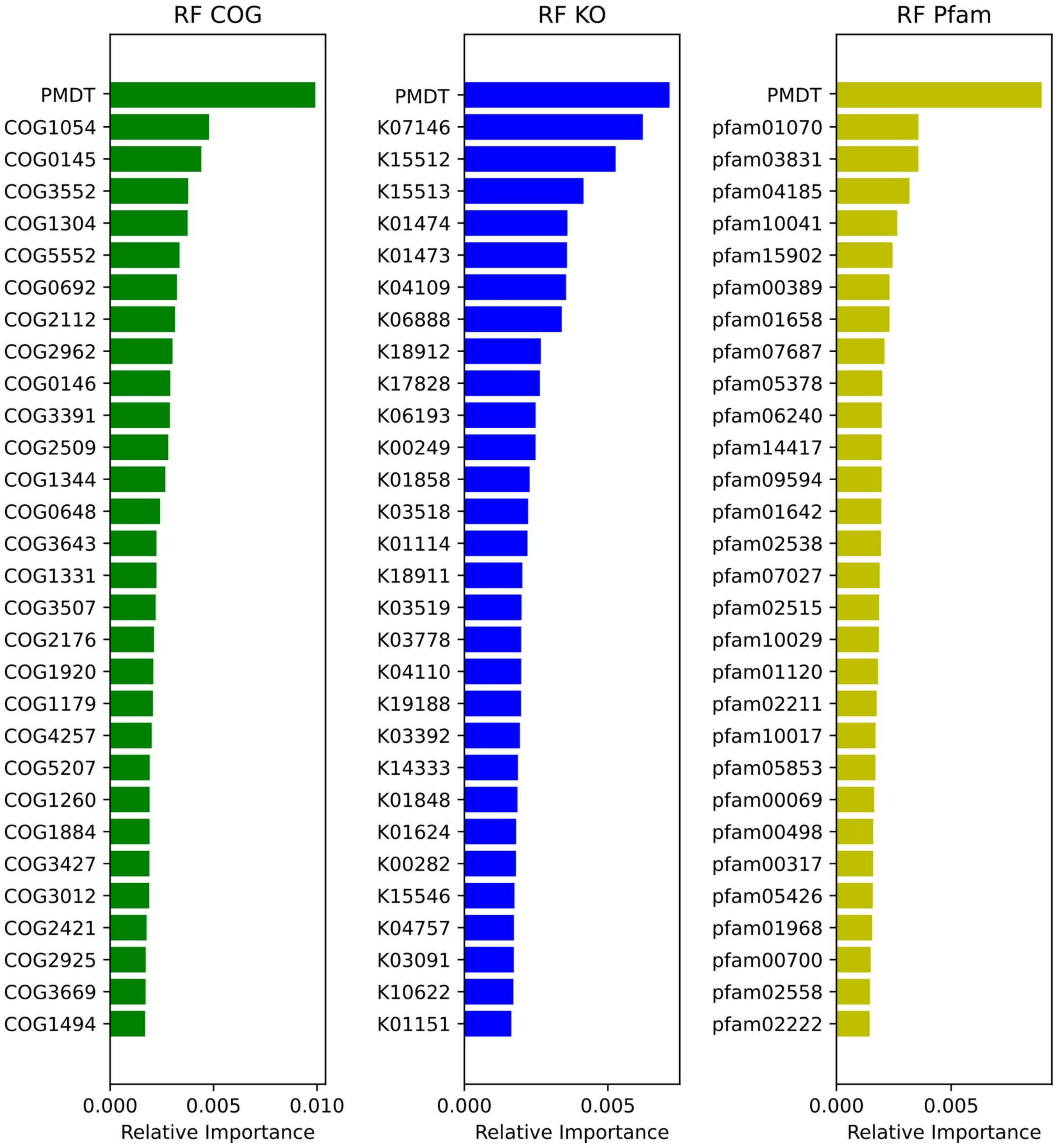

Supplementary Figure 16. Feature importance of RF models based on COG, KO, and Pfam. Models were trained with optimal parameters and their 30 most important features are displayed.

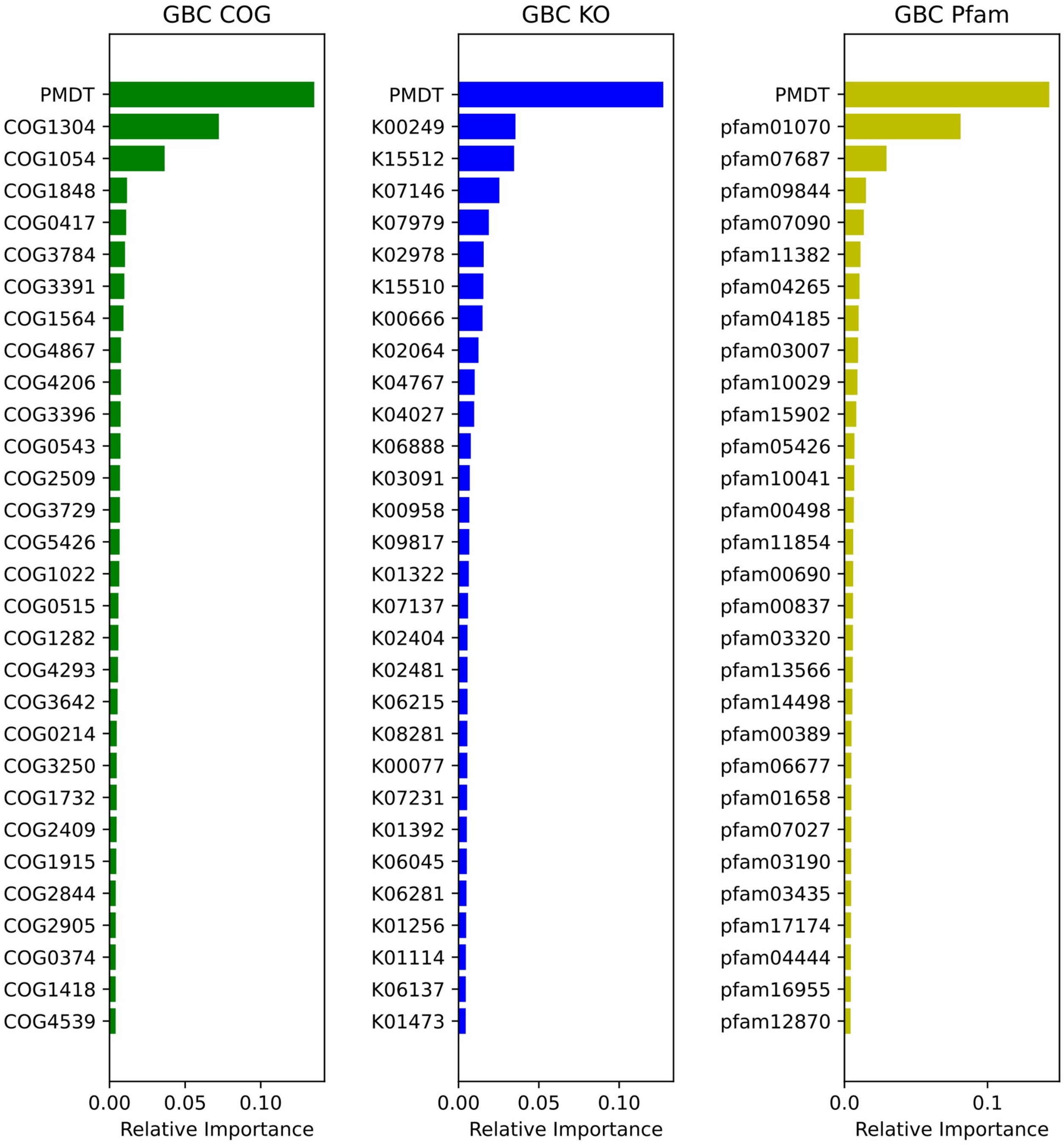

Supplementary Figure 17. Feature importance of GBC models based on COG, KO, and Pfam. Models were trained with optimal parameters and their 30 most important features are displayed.

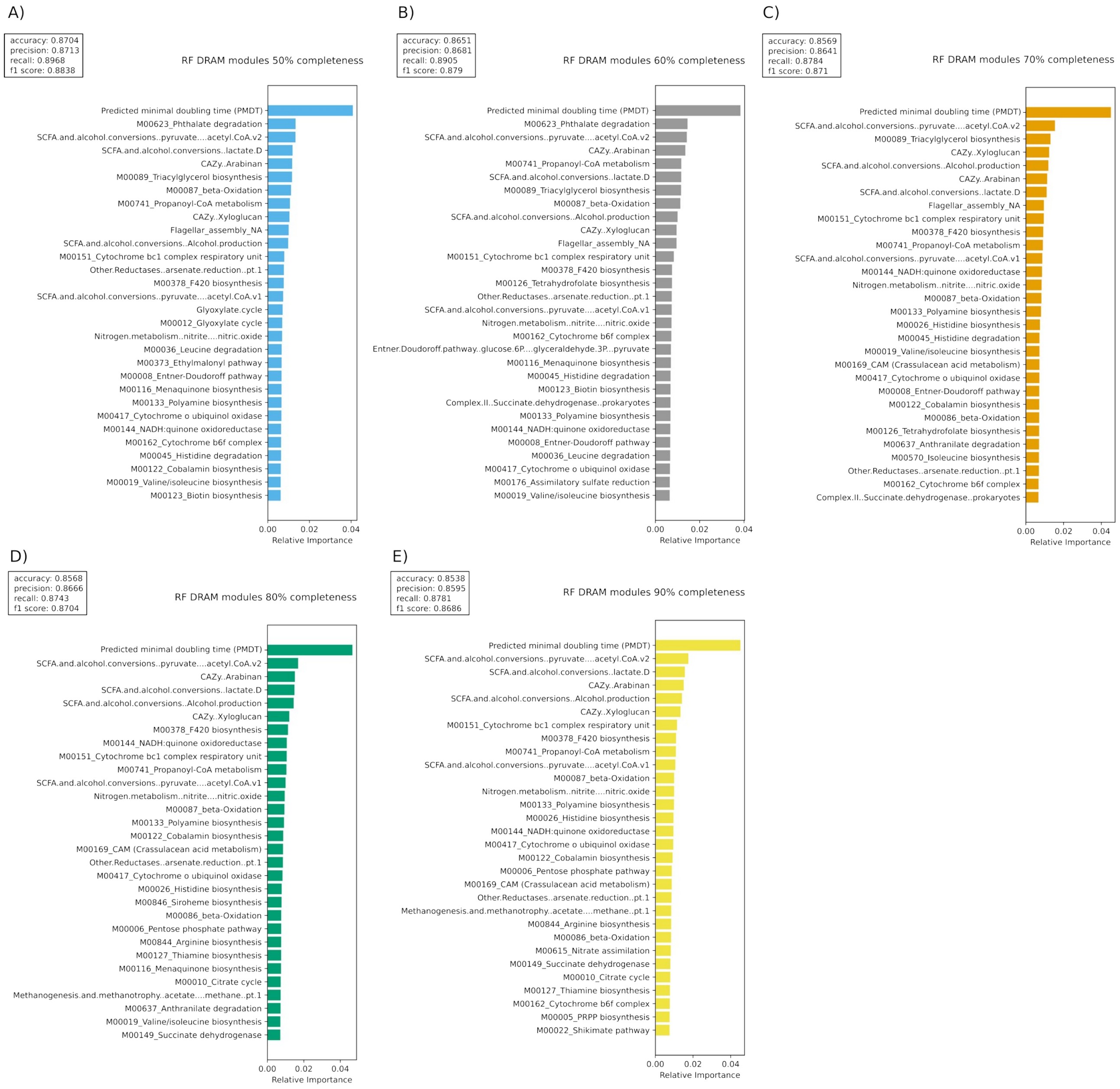

Supplementary Figure 18. Feature importance of RF models based on DRAM modules. Models were trained with default parameters and n_estimators = 300. Their 30 most important features are displayed along with their annotations. In all cases PMDT is the most important feature, comparing with a wide variety of metabolic modules, as well as CAZy enzymes, or flagella presence. Models were run using binary matrices of the presence or absence for each functional category. A MAG is considered to contain a functional category when module completeness was higher than 50, 60, 70, 80, or 90%, in panels A) to E), respectively.

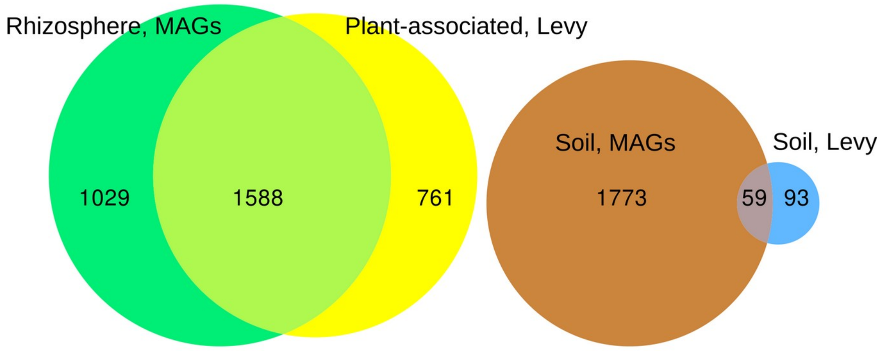

Supplementary Figure 19. Significant COGs found in our work and in Levy et al. Numbers correspond to COGs found significantly associated with rhizosphere and soil bacteria in our work (green and brown) and in Levy's work (yellow and blue), respectively. From 41240 COG-based PhyloGLM tests performed in our work (all COGs in each of the 11 taxonomic groups defined here), 2617 COGs were uniquely associated to rhizosphere MAGs, and 1832 uniquely to soil MAGs. Similarly, numbers from Levy's work correspond to COGs significantly associated to uniquely plant-associated genomes or uniquely to soil genomes with any of the 5 statistical tests done by the authors, with 2349 COGs associated to plant-associated genomes, and 152 associated to soil genomes.
